# C8J_1298, a bifunctional thiol oxidoreductase of *Campylobacter jejuni*, affects Dsb (disulfide bond) network functioning

**DOI:** 10.1371/journal.pone.0230366

**Published:** 2020-03-23

**Authors:** Anna Marta Banaś, Katarzyna Marta Bocian-Ostrzycka, Maciej Plichta, Stanisław Dunin-Horkawicz, Jan Ludwiczak, Jagoda Płaczkiewicz, Elżbieta Katarzyna Jagusztyn-Krynicka

**Affiliations:** 1 Department of Bacterial Genetics, Institute of Microbiology, Faculty of Biology, University of Warsaw, Warsaw, Poland; 2 Laboratory of Structural Bioinformatics, Centre of New Technologies, University of Warsaw, Warsaw, Poland; 3 Laboratory of Bioinformatics, Nencki Institute of Experimental Biology, Warsaw, Poland; 4 Department of Virology, Institute of Microbiology, Faculty of Biology, University of Warsaw, Warsaw, Poland; University of Nebraska-Lincoln, UNITED STATES

## Abstract

Posttranslational generation of disulfide bonds catalyzed by bacterial Dsb (disulfide bond) enzymes is essential for the oxidative folding of many proteins. Although we now have a good understanding of the *Escherichia coli* disulfide bond formation system, there are significant gaps in our knowledge concerning the Dsb systems of other bacteria, including *Campylobacter jejuni*, a food-borne, zoonotic pathogen. We attempted to gain a more complete understanding of the process by thorough analysis of C8J_1298 functioning *in vitro* and *in vivo*. C8J_1298 is a homodimeric thiol-oxidoreductase present in wild type (*wt*) cells, in both reduced and oxidized forms. The protein was previously described as a homolog of DsbC, and thus potentially should be active in rearrangement of disulfides. Indeed, biochemical studies with purified protein revealed that C8J_1298 shares many properties with EcDsbC. However, its activity *in vivo* is dependent on the genetic background, namely, the set of other Dsb proteins present in the periplasm that determine the redox conditions. In *wt C*. *jejuni* cells, C8J_1298 potentially works as a DsbG involved in the control of the cysteine sulfenylation level and protecting single cysteine residues from oxidation to sulfenic acid. A strain lacking only C8J_1298 is indistinguishable from the wild type strain by several assays recognized as the criteria to determine isomerization or oxidative Dsb pathways. Remarkably, in C. *jejuni* strain lacking DsbA1, the protein involved in generation of disulfides, C8J_1298 acts as an oxidase, similar to the homodimeric oxidoreductase of *Helicobater pylori*, HP0231. In *E*. *coli*, C8J_1298 acts as a bifunctional protein, also resembling HP0231. These findings are strongly supported by phylogenetic data. We also showed that CjDsbD (C8J_0565) is a C8J_1298 redox partner.

## 1. Introduction

The disulfide bond formation catalyzed by bacterial proteins of the Dsb (disulfide bond) system is a crucial step in the folding process of a protein. Generally, two Dsb proteins, DsbA and DsbC, operating in the same cell compartment, play a distinct role in the protein’s oxidative folding, affecting the structure of relevant substrates. DsbAs introduce disulfides between cysteine residues that are consecutive in the primary sequence of the protein, while DsbCs rearrange incorrect disulfides introduced between cysteine residues (isomerization pathways). In order to fulfill their roles, most DsbAs cooperate with transmembrane DsbBs that transport electrons into quinone molecules, connecting the disulfide bond generation pathway to the electron transport chain. In this way, DsbBs keep DsbAs in the oxidized form. In contrast to DsbAs, DsbCs, which act as reductases/isomerases, are maintained in reduced forms due to the action of their redox partners—transmembrane DsbD proteins. In this case, reducing equivalents (electrons) are transferred from cytoplasmic thioredoxin in a complex multi-stage process. There is a third periplasmic Dsb–DsbG. Like DsbC, DsbG is kept in the reduced form by DsbD, but DsbG is mainly involved in protecting proteins with a single cysteine residue against inappropriate oxidation. Most of the Dsbs responsible for disulfide bond generation are monomeric proteins, while DsbC and DsbG are homodimers. Two dimeric thiol oxidoreductases of Gram negative bacteria, which function in the oxidative Dsb pathway, have also been described (*Helicobacter pylori* HP0231 and LpDsbA2 of *Legionella pneumophila*) [1–4]. A disulfide isomerase, acting in *Proteus mirabilis* cells and atypical in its structure, has recently been characterized. It is a trimeric protein whose function is dependent on a shape-shifting motif [5]. Despite differences in their structures, both DsbAs and DsbCs contain similar catalytic domains–a thioredoxin fold with characteristic CXXC and cis-Pro (cP) motifs. However, these motifs are not identical. Most (75%) of the characterized monomeric DsbAs contain a CPHC catalytic motif paired with VcP, whereas the DsbC active sites include a CGYC motif paired with TcP. EcDsbG contains the CPYC motif paired with TcP. Several excellent reviews discussing details of the *E*. *coli* Dsb network have recently been published; for example see: [6–13].

Of all the DsbCs described to date, the model-microorganism *E*. *coli* EcDsbC is still the best characterized. Structures of some DsbCs have been determined, and they reveal that this class of oxidoreductases are V-shaped homodimers. Each monomer consists of two domains: the C-terminal catalytic domain and the N-terminal dimerization domain, linked with a flexible, helical linker. The large cleft that is formed is the binding site for relevant substrates (misoxidized proteins and the DsbDα domain) [14–18]. The presence of the dimerization domain and hinged linker prevents EcDsbC oxidation by EcDsbB. EcDsbC mutants present as monomers are able to complement an *E*. *coli* Δ*dsbA* null mutation [19, 20]. EcDsbB is a highly specific protein; it can oxidize only reduced EcDsbA. However, an EcDsbB version that is mutated in a short, membrane-localized α-helix, overcomes the need for EcDsbA and is able to interact with EcDsbC in the process of protein oxidative folding, as measured by motility and cadmium sensitivity tests [21].

Even though the Dsb pathways involving EcDsbA and EcDsbC are generally rather separated, they can assist each other, and their activities are dependent upon the redox environment of the periplasm. EcDsbA can generate disulfide bonds between nonconsecutive cysteine residues under certain conditions, as long as no incorrect disulfides are generated [22]. On the other hand, EcDsbC can support EcDsbA’s action by introducing a disulfide bond in a DsbD–independent manner [23]. Additionally, overproduction of Dsb family proteins from plasmids results in loss of the periplasmic redox balance, which alters Dsb protein activities. High levels of EcDsbA expression that change the redox status, are sufficient to partially complement the lack of DsbC [24].

In comparison to DsbA, which is required for folding of many extracytoplasmic proteins, only a limited number of proteins with various numbers of nonconsecutive disulfides have been identified, so far, as targets of DsbC. The list includes, among others, the penicillin insensitive endopeptidase MepA, the ribonuclease RNase I, the acid phosphatase AppA, the endonuclease EndI, the essential β barrel OM lipoptotein LptD, and RcsF, a component of Rcs–regulation of the capsule synthesis phosphorelay system [23, 25–27]. DsbC plays a crucial role in cell protection against envelope oxidative and copper stress [28–30].

The subject of this study was the Dsb (disulfide bond) system of *Campylobacter jejuni*. Campylobacteriosis is now recognized as the most common zoonosis worldwide, and *Campylobacter jejuni* infections are the leading cause of this bacterial diarrheal disease in humans [31]. Many *C*. *jejuni* strains have been isolated from various sources and characterized with regards to their physiological features, as well as pathogenic traits [32]. The genomes of some have been sequenced, and this showed a high degree of diversity among various isolates. The three most commonly used strains of *C*. *jejuni* in basic, as well as applied, research are *C*. *jejuni* NCTC11168, 81–176 and 81116. These strains were isolated from diarrheic patients [33–35]. Their genome sequences were determined. The *C*. *jejuni* 81116 strain is considered to be more genetically stable than the other two strains, due to a smaller number of hypervariable G tracts. [36–38].

The functioning of the Dsb system of *C*. *jejuni* 81116 has so far been poorly understood. The Dsb oxidative pathway of *C*. *jejuni* (CjDsb) seems much more complex than the one operating in commensal *E*. *coli* [2, 39]. The *C*. *jejuni* 81116 oxidative Dsb pathway involved in disulfide bond generation is composed of four enzymes, of which two [CjDsbA1 (C8J_0814) and/or CjDsbA2 (C8J_0811)] are localized in the periplasm, and two others [CjDsbB (C8J_0812) and CjDsbI (C8J_0016)] are anchored in the inner membrane. *In silico* analysis has led to identification of a potential DsbC (C8J_1298) and DsbD (C8J_0565) [2, 40], enzymes that probably operate in the isomerization pathway responsible for the rearranging of nonnative disulfide bonds. The presence of a thioredoxin fold with a CXXC motif, which is a distinctive feature of proteins that are members of thiol oxidoreductases family, was additionally identified in six other extracytoplasmic proteins (C8J_0031, C8J_1047, C8J_1150, C8J_1566, C8J_1567 and C8J_1568). Homologs of two of them (C8J_1047 and C8J_1150) from *C*. *jejuni* NCTC11168, Cj1106 and Cj1207 respectively, are CcmG (cytochrome C maturation) proteins involved in the process of cytochrome c biogenesis [40]. C8J_0031, a small thioredoxin potentially involved in biogenesis of MccA protein, is an atypical sulfate reductase containing a heme-copper active site [40, 41]; C8J_0031 is not present in the proteome of *C*. *jejuni* NCTC11168 [40]. The function of the three remaining proteins, having a thioredoxin fold with a CXXC motif, is unknown; the genes encoding two of them, at least in *C*. *jejuni* NCTC11168, are iron-regulated [42]. S1 Table (Supporting information) presents the list of characterized and potential *dsb* genes in *C*. *jejuni* 81116 and orthologs in other strains.

Here, we report characterization of the *C*. *jejuni* C8J_1298 protein, previously described as DsbC, by phylogentic analysis and by elucidating its function *in vitro* and *in vivo*. Biochemical work with recombinant C8J_1298 demonstrates several properties typical of DsbC proteins. However, our phylogenetic analysis indicates that C8J_1298 belongs to the same family as HP0231, the *Helicobacter pylori* dimeric thiol oxidoreductase with oxidase activity. We show that functioning of C8J_1298 is determined by the genetic background, where the set of other Dsb proteins determines redox conditions of the periplasm. Substantial differences were observed between C8J_1298 function expressed in various mutated *C*. *jejuni* and *E*. *coli* cells. We also showed that C8J_1298 plays a regulatory role with respect to other components of the Dsb system.

## 2. Results

### 2.1. Substrates of Campylobacter Dsb system–*in silico* analysis

We analyzed the proteome of *C*. *jejuni* 81116 with regards to proteins containing cysteine residues, for their location and the number of cysteine residues. We found that 1328 out of 1582 ORFs encode proteins that contain at least one cysteine residue. Based on the presence of putative signal peptides (SPI—specific for periplasmic proteins and SPII—specific for lipoproteins anchored in inner- or outermembrane of the cell) or transmembrane helices, we predicted that 404 of them encode non-cytoplasmic proteins. Among these, 114 that contain signal sequences and potentially localize in the periplasm or in the outer membrane, were classified as exported proteins. Similar to *Escherichia coli* [43], these exported proteins show a unique bias for even numbers of cysteines (S1 Fig), suggesting that many of them may be substrates for oxidative folding. Our analysis also includes proteins with one cysteine residue because they potentially may be targets of the Dsb protein homolog of EcDsbG.

### 2.2. *c8j_1298* genome location and phylogenetic analysis of its product

The periplasmic dimeric thiol oxidoreductase C8J_1298 was the object of the present research. It was previously described as a homolog of EcDsbC [44, 45]. Interestingly, the XX dipeptide from its active CXXC site is identical to that of EcDsbG (CPYC) but different from that of EcDsbC (CGYC). To gain insight into the possible function of C8J_1298, we analyzed its genomic context and found that it may form an operon with the downstream gene *c8j_1299*, encoding a homolog of LptE protein. The EcLptE protein forms a complex with EcLptD and is involved in the export of lipopolysaccharide (LPS) at the surface of the outer membrane [27, 45]. The biogenesis of EcLptD/LptE complex depends on both EcDsbC and EcDsbA [27, 46]. The process of LPS translocation across OM in *C*. *jejuni* cells has not been investigated so far. In contrast to EcLptD which contains four cysteine residues and two non-consecutive disulfide bonds, *C*. *jejuni* LptD homolog (C8J_1196 in *C*. *jejuni* 81116 strain) contains only two cysteine residues. In other *Campylobacter* species, such as *C*. *insulaenigrae*, *C*. *upsaliensis*, *C*. *lari*, *C*. *helveticus* and *C*. *volucris*, genes coding the C8J_1298 and C8J_1299 (LptE) homologs are also arranged in putative operons, suggesting a functional association between these proteins. It must be noted, however, that although C8J_1299 contains two cysteine residues, one of them is probably a part of the signal peptide. However, if C8J_1298 acts in the way similar to EcDsbG the second cysteine residue of C8J_1299 may be a target of C8J_1298.

**Fig 1:**
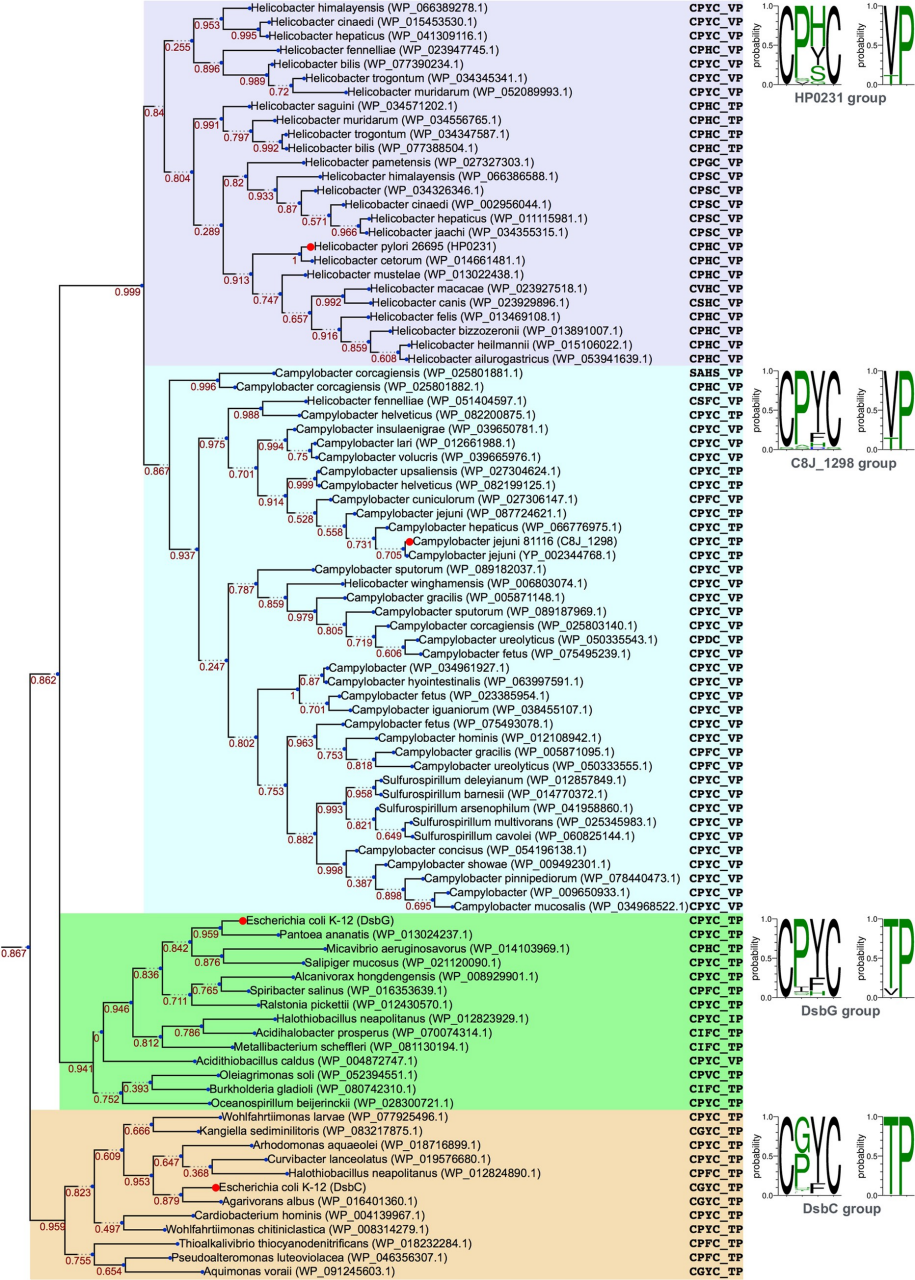
Phylogenetic tree of Dsb catalytic domains. Background colors indicate families: HP0231-like and C8J_1298-like (violet and cyan, respectively), DsbG (green), and DsbC (wheat). Each family is accompanied by a sequence logo representing typical residues in CXXC and XcP motifs (please note that the logos were calculated based on larger data sets). Numbers near branches denote support values. Representative proteins mentioned in the text are indicated with red dots.

The periplasmic dimeric thiol oxidoreductase C8J_1298 belongs to an evolutionarily distinct family encompassing DsbG/C-like proteins from the genera of *Helicobacter*, *Campylobacter* and *Sulfurospirillum*. These proteins are characterized by an N-terminal dimerization domain homologous to the one seen in DsbG and DsbC proteins and by the frequent presence of the VcP cis-proline motif, typical for DsbA proteins (DsbG-like and DsbC-like proteins typically contain a TcP motif). Phylogenetic analysis performed in this study (Fig 1, S1, S2 and S3 Files) suggests that members of this family are most closely related to DsbG-like proteins. This is also supported by the fact that these groups share a common feature of having a nearly invariant proline residue in the second position of the CXXC motif.

The DsbG/C-like family can be subdivided into two subgroups, one comprising *Helicobacter* proteins, including HP0231, which we and others have studied previously [47, 48], and the other comprising C8J_1298 and its homologs from *Campylobacter* and *Sulfurospirillum* species. As mentioned above, most of the HP0231-like and C8J_1298-like proteins have the VcP cis-proline motif. However, in both subgroups, there are also proteins with a TcP cis-proline motif (importantly C8J_1298 and its closest homologs are such exceptions). While the cis-proline motifs are typically similar in the HP0231-like and C8J_1298-like groups, the CXXC motifs are different: HP0231-like proteins contain either a CPYC motif (typical for DsbG proteins) or a CPHC motif (typical for DsbA proteins), whereas the vast majority of C8J_1298-like members, including C8J_1298, contain the CPYC motif.

### 2.3. Biochemical properties of the recombinant C8J_1298 resemble those of members of the DsbC family

To gain insight into C8J_1298 function, we first analyzed the biochemical properties of the protein. Recombinant C8J_1298 (lacking the signal sequence and with His-Tag added on C-terminus of the sequence) used for the biochemical study was obtained from *E*. *coli* as a cytoplasmic protein and purified by affinity chromatography.

**Fig 2:**
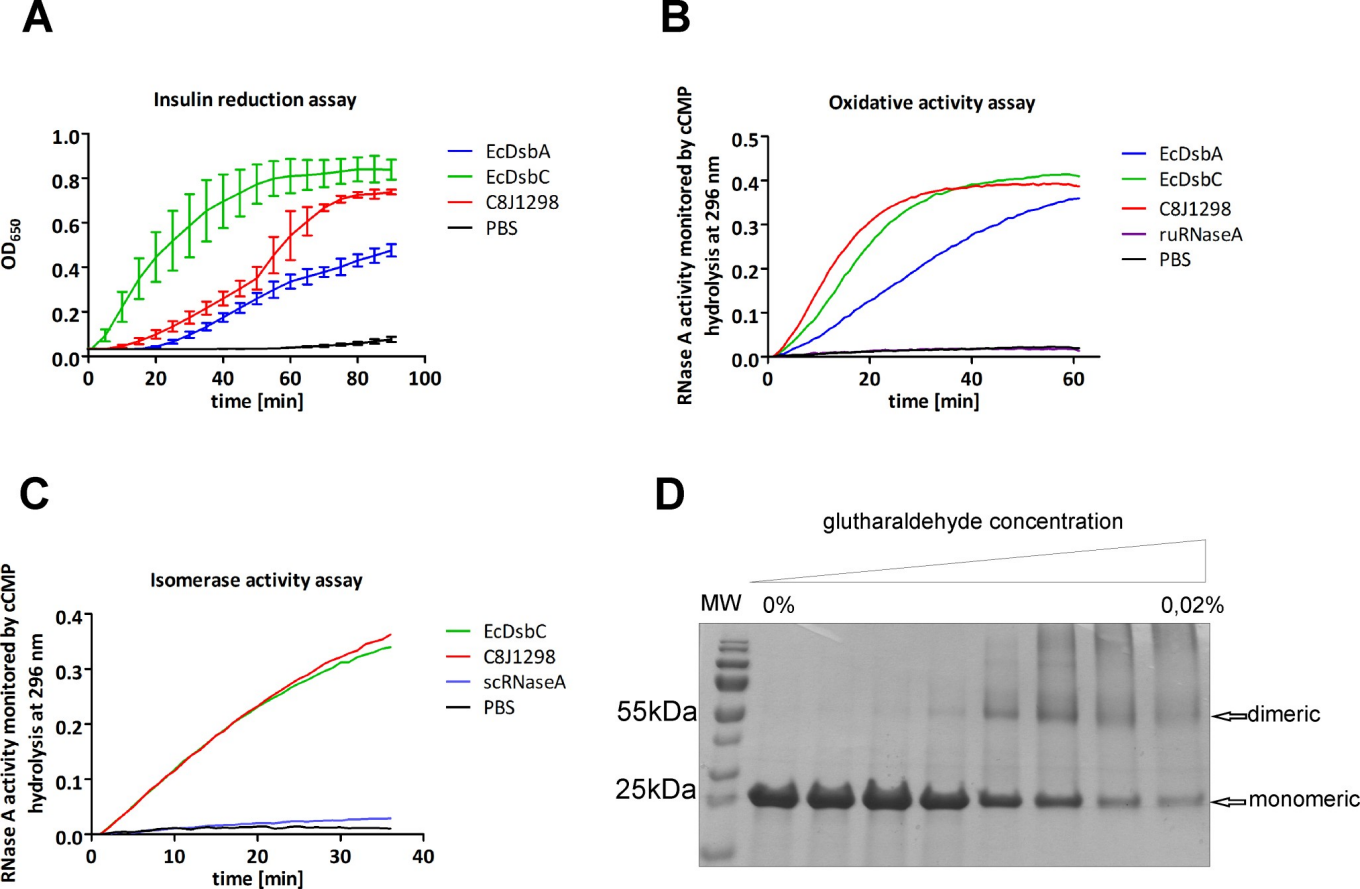
Biochemical features of C8J_1298 resemble those of EcDsbC. Purified EcDsbA and/or EcDsbC were used as controls. (A) Insulin reduction assay. **T**he reaction was performed in the absence (black) or presence of 10 μM of each protein: EcDsbA (blue), EcDsbC (green), C8J_1298 (red). The data presented is the average of three independent experiments, with two technical repetitions (n = 3). (B) *In vitro* C8J_1298 oxidative activity assay using reduced unfolded RNase A (ruRnase A). (C) *In vitro* C8J_1298 isomerase activity assay using scrambled RNase A (scRnase A). (D) Determination of the oligomeric state of C8J_1298 versions using glutaraldehyde. Purified C8J_1298 was cross-linked in the presence of different concentration of glutaraldehyde. Figs B, C, D present representative result based on three independent experiments (n = 3).

First, we evaluated its ability to reduce disulfide bonds using the insulin reduction assay. This assay is commonly used to determine whether a protein can function as an oxidoreductase, regardless of its function in the reducing or the oxidizing pathway *in vivo*. The reduction of intramolecular disulfide bonds between A and B amino acid chains of insulin catalyzed by oxidoreductase is monitored by following the increase of turbidity at 650 nm. We found that C8J_1298 was a really potent factor for this reaction. It catalyzed insulin reduction more efficiently than EcDsbA and almost as effectively as EcDsbC (Fig 2A).

Next, we investigated how efficiently the C8J_1298 can refold reduced ruRNase A (oxidiation activity) or scrambled (improperly folded with wrong disulfides) scRNase A (isomerization activity). In both tests, EcDsbC was used as a positive control. We found that C8J_1298 reactivated RNase A at levels identical to EcDsbC in both assays. Its ability to oxidize cysteine residues of ruRNAse A was even higher than that of EcDsbA (Fig 2B and 2C).

C8J_1298 displayed its isomerizing activity in *in vitro* experiments. As most of the Dsb proteins involved in the isomerization pathway exist as dimers, we also checked the potential oligomerization of C8J_1298 using a glutaraldehyde crosslinking strategy. Glutaraldehyde stabilizes oligomeric proteins by covalent crosslink formation. As shown in Fig 2D the exposure of C8J_1298 to glutaraldehyde resulted in generation of a protein with a molecular weight of approx. 55 kDa, which indicates that C8J_1298 exists as a dimer.

We also determined the C8J_1298 redox potential by equilibrium incubation using GSH/GSSG as a reference. The calculated redox potential for C8J_1298 was -121 mV (S2 Fig), which is similar to that of EcDsbA (-120 mV) or EcDsbG (-127 mV), and slightly higher than that of EcDsbC (-147 mV) [49].

Taken together, the *in vitro* analyses of C8J_1298 suggested its role in the isomerization Dsb pathway, although its redox potential was rather similar to that of EcDsbA.

### 2.4. C8J_1298 is involved in disulfide generation *in vivo* and can substitute for CjDsbA1

Next, in order to provide new insights into the functioning of the *C*. *jejuni* 81116 disulfide bond formation system, we decided to examine C8J_1298 functioning *in vivo*.

**Fig 3:**
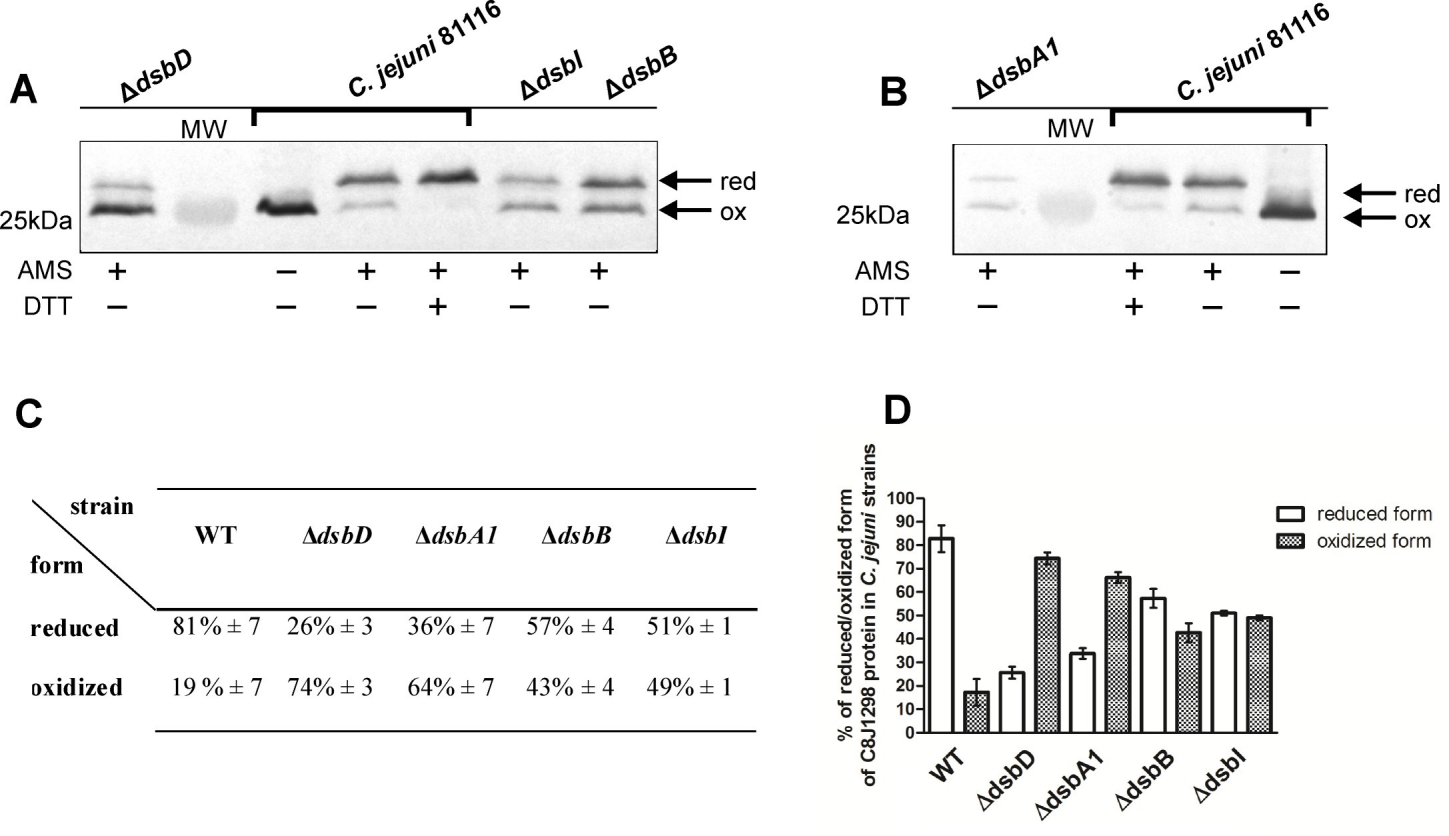
Redox state of C8J_1298 in various strains of *C*. *jejuni* 81116. (A) Strains lacking membrane Dsb proteins: Δ*dsbD* (AB2), Δ*dsbI* (AG4) and Δ*dsbB* (AG3). (B) Strain lacking CjDsbA1 (KBO1). Bacterial cultures were treated with 10% TCA, followed by alkylation with AMS. Cellular proteins including the reduced (red; DTT treated, modified with AMS) and the oxidized (ox; non-modified with AMS) wild type controls were separated by 14% SDS-PAGE under non-reducing conditions, and Western blot analysis using antibodies against C8J_1298 was performed. Each lane contains proteins isolated from the same amount of bacteria. (C) Table and (D) plot presents equlibrium beetwen reduced and oxidized forms of C8J_1298 in various *C*. *jejuni* 81116 strains. Proportion of reduced and oxidized forms of protein was estimated using ImageLab^™^ (Bio-Rad). Result is the average of three independent experiments, with two technical repetitions (n = 3). The figure presents a representative result.

**Fig 4:**
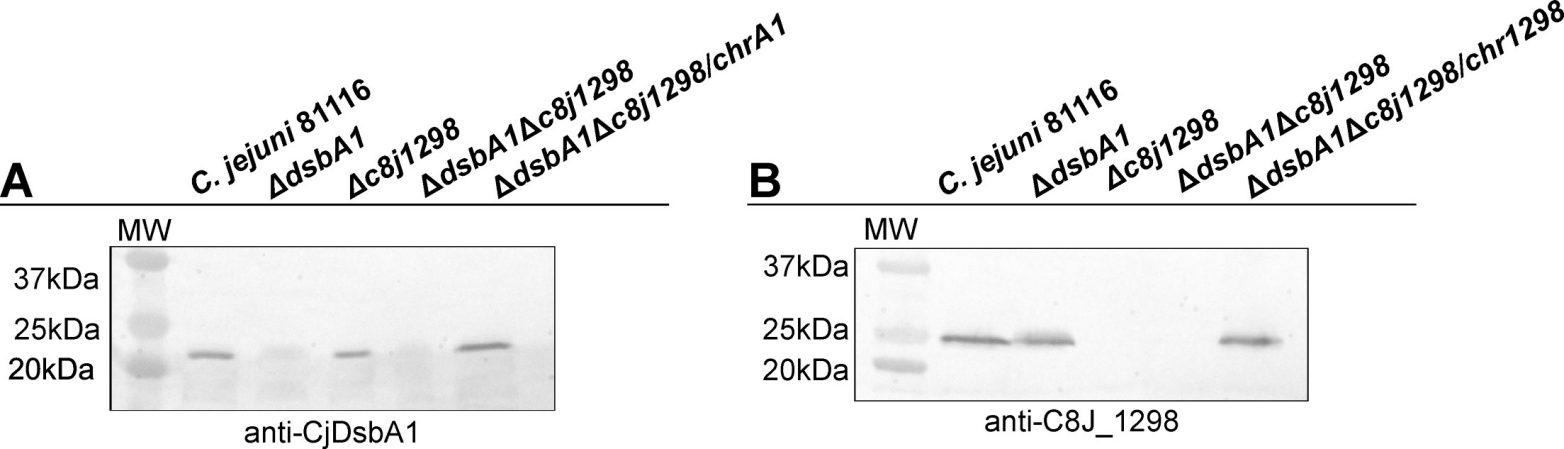
Construction of Δ*dsbA1*, Δ*dsbA1*Δ*c8j_1298* deficient strains and double mutant complemented strains (Δ*dsbA1*Δ*c8j_1298*/chrA1, Δ*dsbA1*Δ*c8j_1298*/chr1298). *C*. *jejuni* proteins (whole cell lysates) were separated by 12% SDS-PAGE and electrotransferred onto nitrocellulose membrane. Specific rabbit sera with antibodies against (A) CjDsbA1 or (B) C8J_1298 were used to verify the absence or presence CjDsbA1 or C8J_1298 in *C*. *jejuni* 81116 cells, respectively.

As the first stage of studying C8J_1298 function *in vivo*, we determined its redox state. The *in vivo* redox states of the Dsb proteins reflect their function. Those involved in disulfide formation, and in most cases reoxidized by DsbB, are present in the oxidized forms, while those active in the reduction/isomerization pathway are kept in reduced forms by DsbD or its shorter version, CcdA [12, 50]. The redox state of C8J_1298 was evaluated by the commonly used AMS-trapping technique which enables the separation of the oxidized and reduced forms of the Dsb proteins by non-reducing SDS-PAGE [51]. The redox state of C8J_1298 was determined in *wt* and in *c8j_0565* (C8J_0565 is a homolog of EcDsbD) mutated cells. The *C*. *jejuni* strain lacking *CjDsbD* (AB2) was generated by allelic exchange technology using the pUWM1512 recombinant plasmid containing the *CjDsbD* gene disrupted by insertion of a chloramphenicol resistance cassette into the gene coding sequence. We found that in *wt* cells, most of C8J_1298 (81%) was present in the reduced form. In cells lacking CjDsbD (AB2), C8J_1298 appeared also in two forms; however the majority of this protein (74%) was present in the oxidized form (Fig 3A, 3B and 3C). The experiment showed that CjDsbD is a redox partner of C8J_1298, responsible for its reduction. The redox state of C8J_1298 was also determined in *C*. *jejuni* strains lacking other *dsb* genes [*CjdsbA1* mutant (KBO1), *CjdsbB* mutant (AG3) and *CjdsbI* mutant (AG4)]. The AG3 and AG4 strains were generated previously (39). The *C*. *jejuni* strain lacking *CjdsbA1* Cm^r^ (KBO1) was constructed using the pUWM1458 recombinant plasmid containing the *CjdsbA1* gene disrupted by insertion of a chloramphenicol resistance cassette into the gene coding sequence. The correctness of this genetic manipulation was confirmed by PCR and by Western blot analysis with rabbit polyclonal anti-DsbA1 antibodies (Fig 4A). In contrast to previously constructed *C*. *jejuni* Δ*CjdsbA1* Km^r^ (AG1) mutant, the new mutated strain was motile [39]. To clarify this inconsistency we performed several genetic experiments and found that the previously observed lack of motility of the Δ*dsbA1* strain was a consequence of background mutation/s (for more details see supporting information S1 Text). All described mutations (Δ*dsbD*, Δ*dsbI*, Δ*dsbB*, Δ*dsbA1****)*** eliminated the preponderance of the reduced form of C8J_1298 when compared to *wt* cells (Fig 3).

To shed more light on the role of C8J_1298 in cell physiology, an isogenic *c8j_1298* knock-out *C*. *jejuni* strain was generated by allelic exchange strategy using a recombinant plasmid unable to replicate in host cells and containing the *c8j_1298* gene disrupted by a kanamycin resistance cassette (AB1). Correct construction of the plasmid was verified by PCR with appropriate pair of primers and by DNA sequencing. The expected inactivation of the chromosomal *c8j_1298* locus as a result of the double cross-over recombination event was verified by PCR using appropriate pairs of primers. The loss of the *c8j_1298* gene product was also confirmed by Western blot of whole-cell proteins with polyclonal rabbit anti-C8J_1298 antibodies (S3A Fig). Generation of the mutation in *c8j_1298* gene does not affect the transcription of *c8j_1299* (S4 Fig). To study the impact of the lack of C8J_1298 on cell physiology, two complemented strains of the *c8j_1298* mutant were generated; one contained the *c8j_1298* gene with its own promoter inserted into the *c8j_0049* pseudogene (Δ*c8j_1298*/chr1298). Correct *c8j_1298* gene integration into the pseudogene was confirmed by PCR. The second strain harbored a recombinant plasmid (pUWM1501), which is derivative of pRY111 able to replicate in *C*. *jejuni* as well as in *E*. *coli*. It contains the *c8j_1298* gene expressed from its own promoter (Δ*c8j_1298*/pl1298). The starting point for pRY111 was the low-copy number, shuttle cloning vector pIL550 with two replication systems derived from pBR322 and pIP1433 (*Campylobater* cryptic plasmid) [52, 53] In both cases, the correct construction of the complemented strains was verified by Western blot analysis with anti-C8J_1298 serum (S3A Fig). However when we tested the presence of CjDsbA1 (a thiol oxidoreductase of oxidative activity) in the proteome of a *c8j_1298 in trans* complemented strain, we found the unexpected loss of this disulfide generating enzyme from the strain proteome (S3B Fig). We also found that this strain does not produce CjDsbA1 transcript (S5 Fig). Finally, to clarify these results, we analysed the *dsbA1* gene in all the strains utilized by PCR and DNA sequencing. We found that the genomes of all strains harbouring the recombinant plasmid with *c8j_1298* contained a *dsbA1* gene that was about 0.8 kb longer than native *dsbA1*. Thus, these complemented strain were excluded from further experiments.

To study the interplay between DsbA1 and C8J_1298 the Δ*c8j_1298*Δ*dsbA1* strain was also generated by allelic exchange technology by introducing a *CjdsbA1* deletion (using suicide plasmid pUWM1458) into the Δ*c8j_1298* strain (AB1). To confirm double Δ*c8j_1298*Δ*dsbA1* mutation effect on cell physiology the two complemented strains of the Δ*c8j_1298*Δ*dsbA1* mutant were generated; they contained the *c8j_1298* or *CjdsbA1* genes with their own promoter sequences inserted into the *c8j_0049* pseudogene, generating Δ*c8j_1298*Δ*dsbA1*/chr1298 and Δ*c8j_1298*Δ*dsbA1*/chrA1, respectively. The correctness of this genetic manipulation was also confirmed by PCR reaction and by Western blot analysis with rabbit polyclonal anti-C8J_1298 and anti-DsbA1 antibodies (Fig 4).

**Fig 5:**
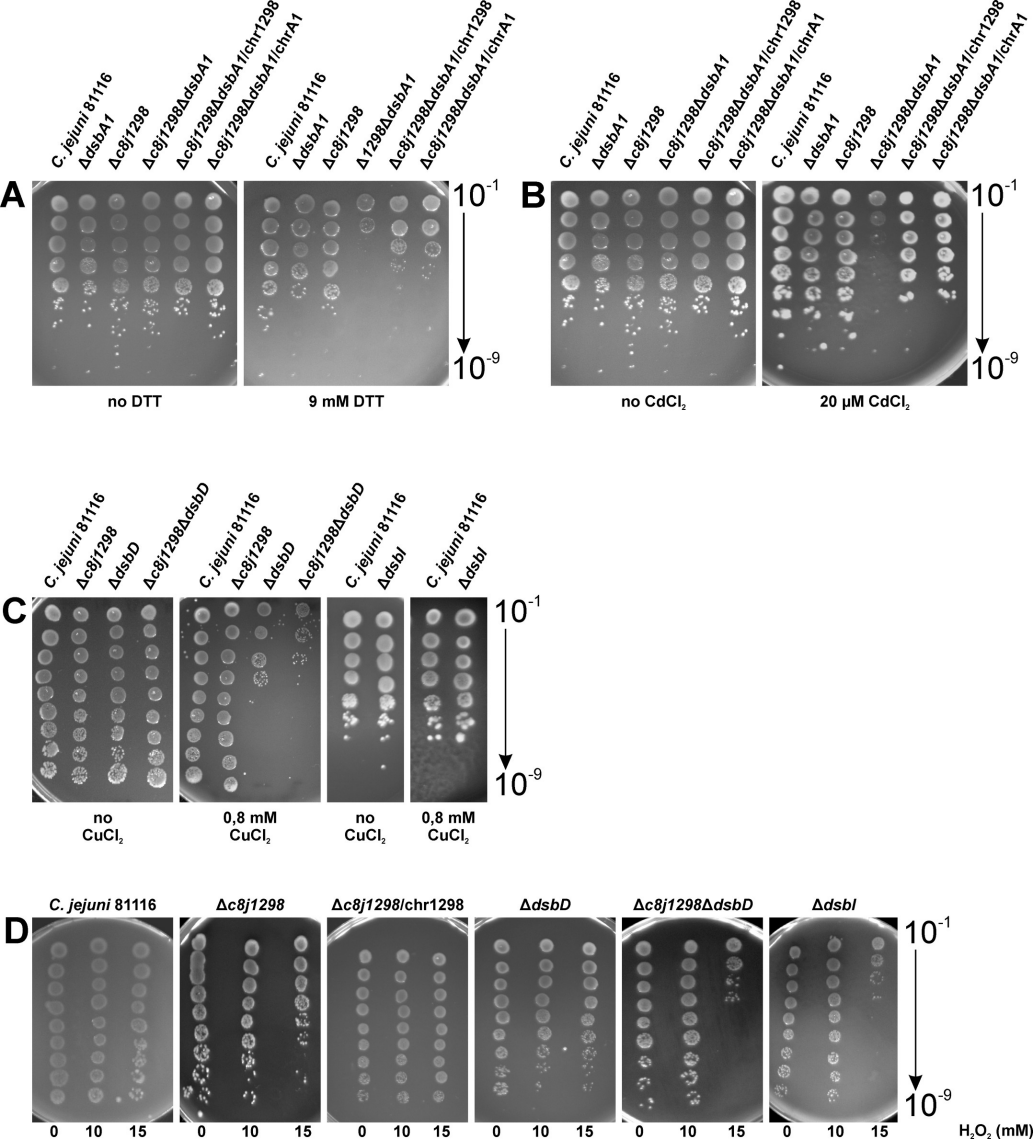
Impact of the lack of C8J_1298 on various cell physiological properties measured by spot-titer method. *C*. *jejuni* 81116 strains in exponential growth phase were 10-fold diluted and spotted on BA plates with DTT (A), CdCl_2_ (B) or CuCl_2_ (C). (D) Viability assay after exposure on hydrogen peroxide was performed with *C*. *jejuni* 81116 strains. Bacterial cultures in exponential growth phase were incubated with hydrogen peroxide one hour, then 10-fold diluted and spotted on BA plates. Three independent experiments with two technical repetitions were performed (n = 3). The figures present a representative result.

Based on our biochemical data, we initially attempted to determine the role of C8J_1298 in the reducing Dsb pathway (DsbC vs DsbG). However, as the catalytic domain of C8J_1298 appeared to belong to the same family as HP0231 *Helicobacter pylori* (a homodimeric Dsb protein functioning in oxidative pathway) [1, 48, 54] we couldn’t exclude the possibility that C8J_1298 was involved in disulfide bond generation. Thus, we evaluated influence of the lack of C8J_1298 on various cell physiological properties recognized as determinants of the Dsb oxidative or isomerization pathways. First, we determined the sensitivity of mutated cells to the reducing agent DTT and to cadmium, as both properties are considered as phenotypic traits of proper Dsb oxidative pathway functioning. Cadmium binds efficiently to free thiol groups of proteins. Thus, cells deficient in introducing disulfide bonds are extremely cadmium sensitive [49, 55]. The DTT and cadmium assays with *wt*, Δ*CjdsbA1* (KBO1), Δ*c8j_1298* (AB1) and Δ*CjdsbA1*Δ*c8j_1298* (AB4) double-mutated strains were performed by the spot-plating method. The results of these experiments are presented in Fig 5A and 5B. We found that the lack of C8J_1298 or DsbA1 only slightly increased the cell sensitivity to both reagents when compared to *wt*. Very interestingly, the double-mutated strain, lacking DsbA1 and C8J_1298, revealed a markedly enhanced sensitivity to DTT and cadmium. Thus, our data indicated that both thiol oxidoreductases, CjDsbA1 and C8J_1298, function in protein oxidative folding and can functionally substitute for each other. This hypothesis is consistent with the results of the chromosomal complementation experiments of both the *c8j_1298* and *CjdsbA1* genes (Δ*c8j_1298*Δ*dsbA1*/chr1298 and Δ*c8j_1298*Δ*dsbA1*/chrA1, respectively). The phenotype was partially corrected (return to lack of sensitivity to DTT or cadmium) irrespective of which mutation, *CjdsbA1* or *c8j_1298*, was complemented (Fig 5A and 5B).

Given the *in vitro* ability of C8J_1298 to catalyze disulfide rearrangements with scRNase A as a substrate, we next evaluated the copper sensitivity of mutated cells. Copper is a nonspecific thiol oxidant that introduces incorrect disulfides. Thus, cells lacking DsbC, which is a key factor for nonnative disulfides rearrangement, are copper sensitive. This feature is DsbD (redox partner of DsbC) dependent [11, 28]. The assay showed that cells lacking C8J_1298 were not impaired in copper toxicity when compared to *wt*. In contrast, cells mutated in *CjdsbD* (AB2) were extremely sensitive to copper. Double-mutated cells lacking both proteins, C8J_1298 and CjDsbD (AB3), revealed a slightly higher sensitivity than the strain lacking only CjDsbD (Fig 5C). The data implied that an oxidoreductase other than C8J_1298, and the target of a potential CjDsbD, plays a key role in the defense against copper stress, and that the role of C8J_1298 is only auxiliary.

*C*. *jejuni* is an oxygen-sensitive microaerophilic microorganism which must combat oxidative stress encountered in the host and in the environment. Various defense mechanisms acting in the cytoplasm of this microorganism have been characterized in detail [56, 57]. Disulfide isomerase DsbC of *E*. *coli* is a periplasmic factor involved in protection against oxidative stress [29]. Thus, we decided to examine the role of the *C*. *jejuni* Dsb system in the defense against oxidative stress. Specially, we wondered whether C8J_1298 participates in this process. It should be noticed that due to the differences in the stress regulation mechanisms, *C*. *jejuni* 81116 exhibits greater resistance to peroxide stress than strains 11168H and 81–176 [58]. We compared the sensitivity to oxidative stress, induced by H_2_O_2,_ of *wt*, Δ*c8j_1298* (AB1), Δ*CjdsbD* (AB2) and *Δc8j_1298ΔCjdsbD* (AB3) mutated strains by determining their survival under oxidative stress conditions. Cells were exposed to H_2_O_2_ for one hour. The assay was performed by the spot plating method. In this experiment, we found that the Δ*c8j_1298* (AB1) and Δ*CjdsbD* (AB2) single mutated strains were only slightly less proficient in protection against oxidative stress in comparison to wt strain (Fig 5D). The Δ*c8j_1298*Δ*CjdsbD* (AB3) double mutant was more sensitive than the single Δ*CjdsbD* mutant (Fig 4D). So, this experiment showed that C8J_1298 is not the main player in the reductive Dsb pathway, and it does not play a key role in defense against oxidative stress or reduction of improper disulfide bonds.

CjDsbI (C8J_0016) protein, which is an atypical CjDsbB-like protein of unknown function, present in many, but not all, members of *Epsilonproteobacteria*, was also included in this analysis because the lack of CjDsbI influences the redox state of C8J_1298 (Fig 3A) [2]. Inspection of the CjDsbI amino acid sequence revealed that this protein comprises two domains. The N-terminal domain of CjDsbI, which consists of five predicted transmembrane segments, exhibits significant sequence similarity to proteins from the DsbB family. In contrast, the C-terminal domain, which is absent from the classical DsbBs, is predicted to locate to the periplasm and fold into a β-propeller structure. CjDsbI contains the CXXC motif located in the 1–2 periplasmic loop. However, it lacks a second pair of Cys residues located in the 3–4 periplasmic loop of classical DsbBs that are involved in interaction with DsbAs [2, 59]. Thus, since it probably does not interact with CjDsbAs, we wanted to test its role in the Dsb reductive pathway that is often involved in defense against oxidative stress. Interestingly, mutation of the *CjdsbI* gene (AG4) resulted in the highest degree of strain sensitivity to peroxide (Fig 5D). To gain more information about DsbI function, we also checked the sensitivity of the Δ*CjdsbI* mutated strain to copper toxicity. We found that cells lacking DsbI were not impaired in copper sensitivity compared to *wt* (Fig 5C). The mechanism of DsbI activity and its interplay with other Dsb proteins still remains to be determined. It is active in a separate Dsb reductive pathway responsible for the defense against oxidative stress but it is not involved in general disulfides rearrangement.

### 2.5. C8J_1298 complements lack of *E*. *coli* DsbA and DsbC proteins

**Fig 6:**
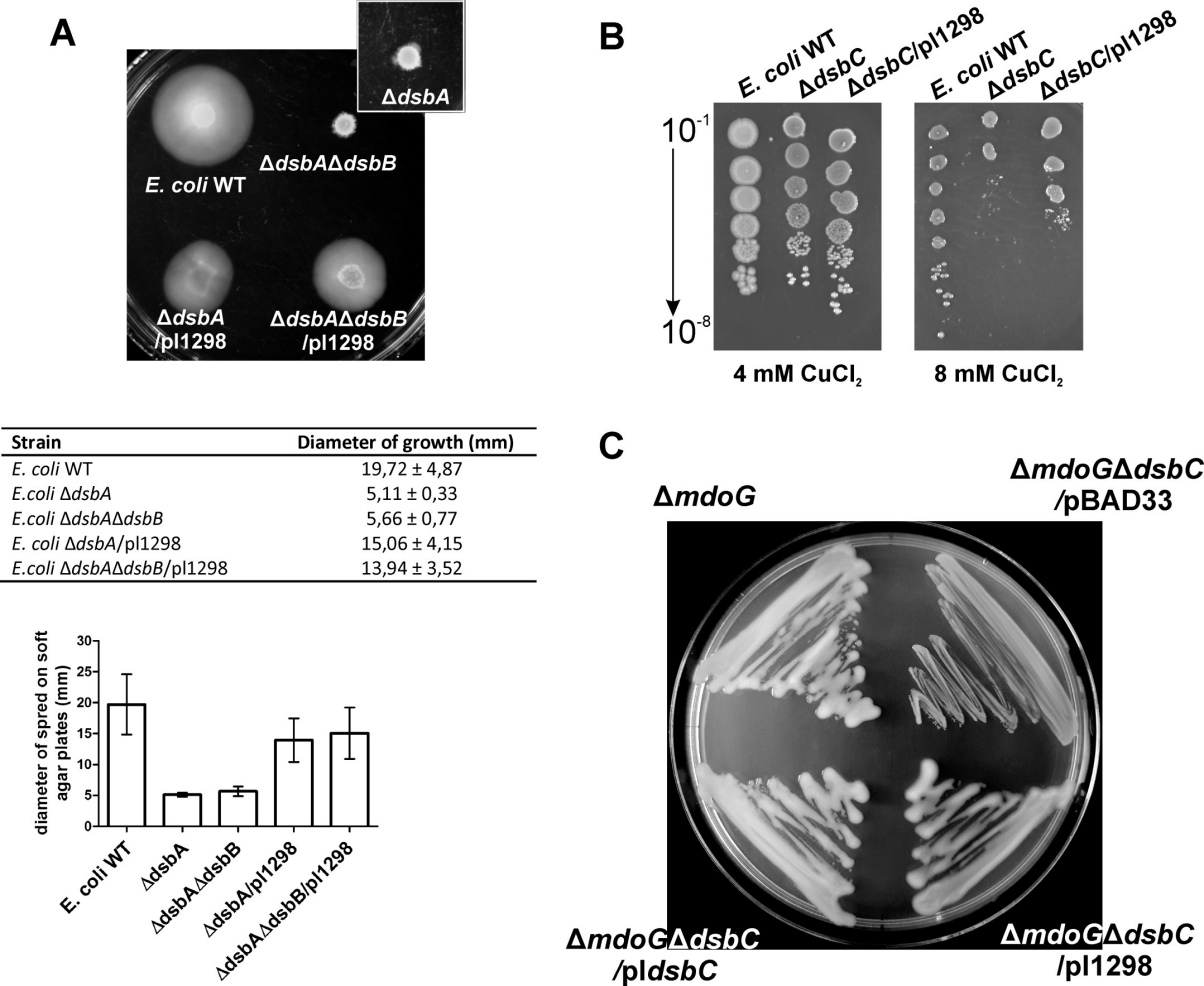
C8J_1298 activity in *E*. *coli dsb* deficient strains. (A) Motility assay: Exponentially growing *E*. *coli* strains were spotted on soft agar plates and incubated 24h at 37°C. C8J_1298 restored motility in both *dsbA* and *dsbAdsbB* deficient strains Table and plot present diameter of growth on soft agar plates of tested strains. Presented average is the result of three independent experiments (n = 3). (B) Copper sensitivity assay: Exponentially growing *E*. *coli* strains were 10-fold diluted and spotted on BHI-agar plates supplemented with arabinose and CuCl_2_. Presence of C8J_1298 only slightly increased resistance to copper toxicity in *E*. *coli dsbC* lacking strain. (C) Mucoid phenotype assay: *E*. *coli* strains were cultivated on M63 minimal medium. C8J_1298 restored mucoid phenotype in *mdoG* deficient *E*. *coli* strain. In all assays three independent experiments were performed (n = 3) and bacterial cultures growing without arabinose supplementation were used as controls. The figures present a representative result.

To further investigate the role of C8J_1298, we evaluated its functioning in *E*. *coli* cells to determine whether it could complement the lack of EcDsbA or EcDsbC. To this end, we cloned *c8j_1298* harboring a DNA fragment encoding the *pelB* signal sequence under an arabinose inducible promoter into a low copy number vector, pMPM-A6 [60]. The construct was chemically transformed into four *E*. *coli* strains: JCB817 Δ*dsbA*, JCB818 Δ*dsbA*Δ*dsbB*, JCF383 Δ*dsbC* and PL263 Δ*mdoG*Δ*dsbC*. The expression of C8J_1298 upon arabinose induction was confirmed by Western blot experiments using specific anti-C8J_1298 antibodies (S6 Fig). As *E*. *coli* flagellar P-ring protein FlgI is one of the substrates of EcDsbA, the mutant lacking DsbA does not produce functional flagella and as a consequence is not motile [61]. We found that C8J_1298 (Δ*dsbA*/pl1298; AB1518) was a folding factor for *E*. *coli* FlgI, as it complemented motility to 70% that of wild type (diameter of spreading growth). However, in contrast to EcDsbA, it functioned independently of EcDsbB (Δ*dsbA*Δ*dsbB*/pl1298; AB1524) (Fig 6A). The processes of complementation are strictly dependent upon the presence of arabinose.

The ability of C8J_1298 to complement EcDsbC was assessed by two strategies. First, we evaluated the copper sensitivity of *E*. *coli* Δ*dsbC* containing the recombinant plasmid expressing C8J_1298 (Δ*dsbC*/pl1298; AB1519) in comparison to *E*. *coli* wild type (JCB816) and to the *E*. *coli* Δ*dsbC* mutant (JCF383). Although none of the tested strains was sensitive to 4 mM CuCl_2_, *E*. *coli* Δ*dsbC*, as expected, exhibited sensitivity to 8mM CuCl_2_. *E*. *coli* expressing C8J_1298 was slightly more proficient in protecting cells from copper toxicity when compared to the Δ*dsbC* mutant; however, it was less proficient than the *wt* strain (Fig 6B). Furthermore, we also examined the influence of C8J_1298 on the oxidative folding and functioning of EcRcsF, a component of the Rcs phosphorelay signal transducing system activated upon envelope stress. EcRcsF is a small outer-membrane lipoprotein oriented towards periplasm. Correctly folded RcsF senses the envelope stress and activates the signaling cascade. As this lipoprotein contains two non-consecutive disulfide bonds, its activity is EcDsbC dependent. An *E*. *coli* Δ*mdoG* mutant, used in this assay, is defective in the synthesis of membrane-derived oligosaccharide and induces the Rcs signaling system in a RcsF-dependent manner [62]. To evaluate the impact of C8J_1298 on EcRcsF oxidative folding, the *E*. *coli* Δ*mdoG*Δ*dsbC* (PL263) mutant was transformed with a recombinant plasmid expressing C8J_1298 under an arabinose promoter (Δ*mdoG*Δ*dsbC*/pl1298; AB1520). An *E*. *coli* Δ*mdoG* mutant exhibits a mucoid phenotype on M63 minimal medium, while a double Δ*mdoG*Δ*dsbC* mutant does not, due to the lack of activation of the Rcs cascade [63]. As presented in Fig 6C C8J_1298 complemented the lack of EcDsbC in this assay, implying that it was able to catalyze disulfide bond rearrangements, at least with regards to EcRcsF. The complemetation process is dependent on the presence of arabinose.

**Fig 7:**
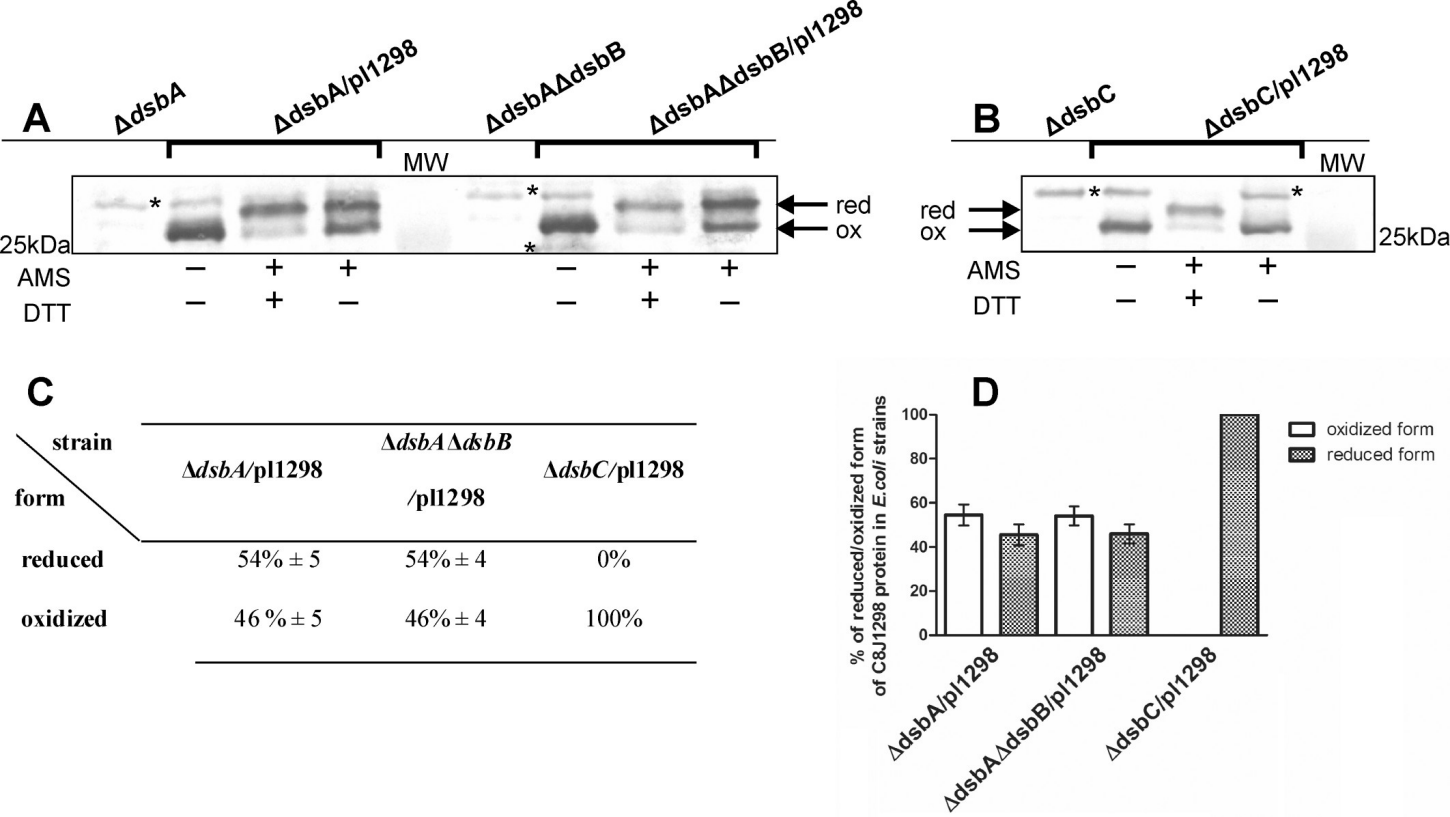
**Redox state of C8J_1298 in (A) *E*. *coli* strains deficient in proteins of oxidative Dsb pathway.** (B) *E*. *coli* strain deficient in EcDsbC protein Bacterial cultures were treated with 10% TCA, followed by alkylation with AMS. Cellular proteins including the reduced (red; DTT treated, modified with AMS) and the oxidized (ox; non-modified with AMS) wild type controls were separated by 14% SDS-PAGE under non-reducing conditions, and Western blot analysis using antibodies against C8J_1298 was performed. Each lane contains proteins isolated from the same amount of bacteria. (C) Table and (D) plot presents equlibrium beetwen reduced and oxidized forms of C8J_1298 in various *E*. *coli* strains. Proportion of reduced and oxidized forms of protein was estimated using ImageLab^™^ (Bio-Rad). Result is the average of three independent experiments (n = 3). (*) unspecific serum reaction. The figure presents a representative result.

Given that C8J_1298 acts in *E*. *coli* as oxidase, as well as an isomerase, we next examined its redox status in *E*. *coli* cells by an AMS/thiol trapping experiment. We found that in *E*. *coli* Δ*dsbA* and Δ*dsbA*Δ*dsbB* strains, C8J_1298 is present in both forms, oxidized as well as reduced. In contrast, in an *E*. *coli* Δ*dsbC* strain, it accumulates in the oxidized state, which suggests that it is a poor substrate for EcDsbD (Fig 7).

## 3. Discussion

The formation of disulfide bonds (Dsb) between cysteine residues stabilizes structure of bacterial extracytoplasmic proteins that play a key role in bacterial virulence. *In silico* analysis indicated that *C*. *jejuni* encodes many exported proteins containing more than two cysteine residues, potential targets of the oxidative Dsb system. Also, some proteins with transmembrane helices localized in the cytoplasmic membrane and having periplasmic loops containing cysteine residues also may be considered as substrates of the Dsb system.

Our previous work has led to characterization of two *C*. *jejuni* thiol oxidoreductases crucial for disulfide generation (CjDsbA1 and CjDsbA2) and their potential redox partner CjDsbB [39]. At the beginning of the present work, we corrected information concerning the impact of the *CjdsbA1* gene product on *C*. *jejuni* cell motility. Through analysis of *C*. *jejuni* genes involved in cell motility [64], in combination with in-depth genetic manipulations (generation of new Δ*CjdsbA1* mutants, Δ*CjdsbA1*Δ*dsbA2* double mutated strains and complementation experiments), we showed that the lack of CjDsbA1 does not influence cell motility. It was documented that various types of mutations (SNPs–single nucleotide polymorphism or INDELs–small insertion and deletions or phase variation changes due to replication error) associated with cell motility and shape occur under standard laboratory conditions and can be introduced at any stage of genetic manipulation [65–68]. Also Pascoe *et al* who analyzed 23 *C*. *jejuni* NCTC11168 strains in respect of their phenotypes and genetic variation in their genomes found that they varied by up to 281 SNPs when compared to reference strain [69]. Although the *C*. *jejuni* 81116 strain is considered to be genetically more stable than two other commonly used strains (NCTC11168 and 81–176), we showed that high caution should be used when the phenotype of a strain associated with generated mutations is evaluated. The previously described *C*. *jejuni* Δ*dsbA1* Km^r^ mutant, apart from the *dsbA1* mutation, harbors extra mutation/s in gene/s potentially related to the flagella assembly process, as this mutant showed an absence of flagella on the cell surface.

The main goal of the present work was to obtain a more complete understanding of how the *C*. *jejuni* oxidative Dsb pathway functions through a thorough analysis of the C8J_1298 periplasmic oxidoreductase of this pathogen. This protein was described as DsbC [44, 45]. However, this kind of designation should always be verified by *in vitro* or/and *in vivo* experiments. For instance, the *Helicobacter pylori* HP0231 dimeric oxidoreductase, which originally was classified as DsbG, turned out to possess both oxidation and isomerization activities [1, 47, 48, 54]. Also, *H*. *pylori* thiol oxidoreductase HP0377 that is a CcmG protein responsible for cytochrome c biogenesis, was described as a DsbC homolog [70].

We took into account two possibilities concerning C8J_1298 function. Based on the facts that C8J_1298 is a homodimeric protein, as shown by glutaraldehyde crosslinking, and that it is kept in the reduced form by CjDsbD, we perfomed several experiments to discriminate between a DsbC and DsbG activity for C8J_1298 [14, 71, 72]. Although *C*. *jejuni* possesses two monomeric homologs of EcDsbA, we have also considered the possibility that C8J_1298 may function as an oxidizing Dsb protein similar to *H*. *pylori* HP0231. The rationale for this hypothesis was our data of phylogenetic analysis that indicated that the catalytic domains of C8J_1298 belongs to the same family as HP0231. Also, the fact that the proportion between oxidized and reduced forms of C8J_1298 are dependent on the presence of other Dsb gene products indicates that this protein may play various roles.

EcDsbC and EcDsbG share only 24% amino acid identity [72]. Both proteins possess superficially similar structures; both are homodimers containing a thioredoxin fold with a CXXC motif (CGYC and CPYC for EcDsbC and EcDsbG, respectively). However, precise comparison of their structures revealed some essential differences, such as the length of the helical linker, the characteristics of the V-shaped cleft and surface charges [72–75]. Thus, they display different biochemical features and generally play various functions in cell physiology. In contrast to EcDsbC, EcDsbG is not able to reduce disulfide bonds, oxidize cysteines or rearrange incorrect disulfide bonds in *in vitro* assays [45, 49, 73, 76]. Our biochemical analysis of the recombinant C8J_1298 demonstrated that C8J_1298, with regard to biochemical features, resembles EcDsbC. One difference between C8J_1298 and EcDsbC is that its redox potential (-121 mV) is more similar to to redox potentials of EcDsbA (-121 mV) and EcDsbG (-127 mV), as opposed to the redox potential of EcDsbC (-143 mV) [49]. However, this assay does not discriminate between various periplasmic thiol oxidoreductases, as they exhibit comparable redox potentials and because the residues in the XX dipeptide between the catalytic cysteines of the conserved CXXC motif have an impact on the redox potential [77, 78].

To verify the data obtained *in vitro*, we performed experiments *in vivo*. Both EcDsbC and EcDsbG are present in *wt* cells in reduced forms, and in oxidized forms in cells lacking DsbD [11]. When EcDsbG is overexpressed, a small amount of protein is present in its oxidized form [76]. In contrast to both EcDsbC and EcDsbG, two forms of C8J_1298 are noticeable in *wt* and in a Δ*CjdsbD* null mutant. The majority of C8J_1298 is in a reduced state in *wt* cells. In contrast, in the Δ*CjdsbD* mutated cells, the majority of protein is in the oxidized state. This data indicates that CjDsbD is a redox partner of C8J_1298. Remarkably, we found that the C8J_1298 functioning *in vivo* is determined by the genetic background, precisely by the set of the Dsb proteins present in the periplasm of the cell. In *wt C*. *jejuni*, C8J_1298 resembled EcDsbG, since it did not show isomerizing or oxidative activity. First, *C*. *jejuni* cells lacking C8J_1298 exhibit almost an identical sensitivity to copper as *wt* cells, which is similar to EcDsbG [28, 73]. However, our data showed that the Dsb reductive pathway is an essential element of this protection system as the Δ*CjdsbD* mutant was noticeably more sensitive when compared to *wt* cells, and double mutated cells lacking two proteins, CjDsbD and C8J_1298, exhibited the highest level of sensitivity. Clearly, *C*. *jejuni* must encode other protein besides C8J_1298 protein, as a CjDsbD partner and a key factor responsible for resolving incorrect disulfide bonds. Details of the process still remain unclear and will require further analysis.

Second, neither a strain lacking DsbA1 nor a strain lacking C8J_1298 is sensitive to DTT or cadmium. However, double mutated cells lacking both, DsbA1 and C8J_1298, are highly sensitive to both reagents, which indicates that both proteins are involved in protein oxidative folding and may substitute for one another. This hypothesis is supported by the fact that the phenotype of the double mutant was partially corrected by chromosomal integration of a functional *CjdsbA1* or *c8j_1298* gene, and it fits well with phylogenetic data that indicates that C8J_1298 is closely related to HP0231. At this point, it should be noted that *H*. *pylori* does not encode classical DsbA and HP0231 is its only factor responsible for disulfide bond generation. We hypothesize that these two redundant genes (*CjdsbA1* and *c8j_1298*) may be differently regulated, that ensures adaptation to various environmental conditions. Our data also showed that in *E*. *coli* the function of C8J_1298 is not restricted to disulfide bond generation; it may be also act as a Dsb isomerase, against selected substrates. Thus, in both *E*. *coli* cells and in *C*. *jejuni* lacking DsbA1, C8J_1298 demonstrates activity similar to *H*. *pylori* dimeric oxidoreductase HP0231 [1, 79].

The observed genetic rearrangements when the *c8j_1298* mutation was *in trans* complemented indicated that maintenance of redox homeostasis is important for cell functioning, and its disturbance results in specific genomic rebuilding. This hypothesis is in accordance with the fact that all our attempts to complement *dsbA1*/*dsbA2* mutation by *trans* complementation were unsuccessful. The situation seems to be exceptionally complicated in microorganisms possessing complex Dsb system with redundant Dsb proteins. An *EcdsbA* mutation can be easily complemented *in trans*, whereas expression from plasmid *Serratia marescens dsbA1* (but not *dsbA2*) or *dsbA1*, *dsbA2* or *c8j_1298* of *C*. *jejuni* have a deleterious impact on cell growth or results in genetic rearrangements [80].

Our analysis of the mechanism of the oxidative stress defense also indicates the complicated interplay between periplasmic Dsb proteins. Activity of CjDsbD, which catalyzes the transfer of electrons across the inner membrane from the cytoplasm to the periplasm, is necessary for the proper course of the defense. However, the obtained data again suggested that C8J_1298 is not the sole CjDsbD target. Interestingly, the most sensitive strain appeared to be that lacking the membrane CjDsbI protein that is an atypical DsbB protein. EcDsbB contains two pairs of conserved, redox-active disulfide bonds. One disulfide is formed between cysteine residues located in the first periplasmic loop, and the second disulfide, which is involved in the initial interaction with reduced EcDsbA, is formed between cysteine residues of the second periplasmic loop [81]. Both essential catalytic cysteine residues of EcDsbB are maintained in an oxidized state [82]. CjDsbI contains only one pair of cysteine residues, located in the first periplasmic loop, and their redox state is unknown [59]. A DsbI mutant did not reveal copper sensitivity, which suggests that this enzyme is an element of the Dsb pathway separate from that responsible for disulfide rearrangements.

In conclusion, we showed that C8J_1298 plays an important role in *C*. *jejuni* Dsb system, and its functioning is dependent upon the genetic background. This homodimeric oxidoreductase acts mainly as an oxidase similar to HP0231 of *H*. *pylori*. The presence of two redundant genes involved in generation of disulfides *(cjdsbA1* and *c8j_1298*) may ensure *C*.*jejuni* to adapt to various environmental conditions. However, it cannot be excluded that C8J_1298, a redox partner of CjDsbD, potentially may be responsible for protecting single cysteine residues against oxidation, as it is not active in cell protection against copper toxicity and oxidative stress. Identification of the C8J_1298 substrates should help to clarify this issue.

## 4. Materials and methods

### 4.1. Bacterial strains, primers, plasmids, media and growth conditions

Bacterial strains, plasmids and primers used in this study are listed in Tables 1 and 2 and S2 in the Supporting information. *Campylobacter jejuni* 81116 strains were grown on Blood Agar base no. 2 (BA) plates (GenoPlast) supplemented with 10% (v/v) horse blood (ProAnimali) or on Mueller Hinton Agar (MH), at 37°C under microaerobic conditions that were provided by Anoxomat Mark II OP (MART® Microbiology B.V) or CampyGen (ThermoFisher Scientific). Liquid cultures were performed on BHI-SG (Brain Heart Infusion supplemented with 20 mM serine and 20 mM glutamine) or MH-S (Mueller Hinton Broth supplemented with 20 mM serine). All media were supplemented with Campylobacter Selective Supplement Blaser-Wang (ThermoFisher Scientific). For the selection of *C*. *jejuni* mutated and complemented strains, kanamycin (30 μg ml^–1^), chloramphenicol (15 μg ml^–1^) or/and spectinomycin (50 μg ml^–1^) were added to the growth media. *E*. *coli* strains were grown at 37°C on solid or liquid Luria-Bertani (LB) medium, BHI agar or on M63 minimal medium [28]. When needed, media were supplemented with antibiotics at the following concentrations: 100 μg ml^–1^ ampicillin, 30 μg ml^–1^ kanamycin, 20 μg ml^–1^ chloramphenicol or/and spectinomycin 50 μg ml^–1^), arabinose (0.2% v/v), X-Gal (13 mg ml^-1^) and/or IPTG (3 mg ml^-1^) in DMF (dimethyl-formamide).

**Table 1:** Strains used in this study.

Name	Relevant characteristics	Source/Ref.
***Escherichia coli* STRAINS**		
**TG1**	*supE44 hsd*Δ *5 thi* Δ(*lac*‾ *proAB*) F’ [*traD36 proAB*^*+*^ *lacI*^*q*^ *lacZ*Δ*M15*]	[83]
**Rosetta(DE3) pLysS**	F‾ *ompT hsdS*_*B*_(r_B_‾m_B_‾) *gal dcm* (DE3) pLysS RARE (Cm^R^)	Novagen
**JCB816**	MC1000 *phoR* λ102	[84]
**JCB817**	MC1000 *phoR* λ102 *dsbA*::*kan1*	[84]
**JCB818**	MC1000 *phoR* λ102 *dsbA*::*kan1*, *dsbB*::*kan*	[84]
**JFC383**	JCB816 *dsbC*::*kan*	JFC collection
**PL260**	MC1000 *mdoG*::*kan1*	[63]
**PL263**	MC1000 *mdoG*::*kan1; dsbC*::*kan2*	[63]
**PL284**	PL263 carrying pBAD33 Km^R^ Cm^R^	[63]
**PL285**	PL263 carrying pJFC355 (*dsbC*^+^ in pBAD33) Km^R^ Cm^R^	[63]
**BL21/*EcdsbA***	BL21 carrying *EcdsbA*^+^ in pET28a Km^R^	JFC collection
**BL21/*EcdsbC***	BL21 carrying *EcdsbC*^+^ in pET28a Km^R^	JFC collection
**KBO1436**	Rosetta(DE3) pLysS carrying pUWM1430 (*CjdsbA1*^+^ in pET28a) Km^R^ Cm^R^	this study
**KBO1441**	Rosetta(DE3) pLysS carrying pUWM1432 (*CjdsbA2*^+^ in pET24a) Km^R^ Cm^R^	this study
**AB1463**	Rosetta(DE3) pLysS carrying pUWM1462 (*c8j_1298*^+^ in pET24a) Km^R^ Cm^R^	this study
**AB1518 (Δ*dsbA*/pl1298)**	JCB817 carrying pUWM1514 (*ss’pelB-c8j_1298*^+^ in pMPMA6) Km^R^ Ap^R^	this study
**AB1519 (Δ*dsbC*/pl1298)**	JCF383 carrying pUWM1514 (*ss’pelB-c8j_1298*^+^ in pMPMA6) Km^R^ Ap^R^	this study
**AB1520 (Δ*mdoG*Δ*dsbC*/pl1298)**	PL263 carrying pUWM1514 (*ss’pelB-c8j_1298*^*+*^ in pMPMA6) Ap^R^ Km^R^	this study
**AB1524 (Δ*dsbA*Δ*dsbB*/pl1298)**	JCB818 carrying pUWM1514 (*ss’pelB-c8j_1298*^+^ in pMPMA6) Ap^R^ Km^R^	this study
***Campylobacter jejuni* 81116 STRAINS**		
**wild type—81116 (NCTC11828)**	parental strain	[33]
**Δ*dsbA1*[old] (AG1)**	*CjdsbA1*::*kan*	[39]
**Δ*dsbA1*[new] (KBO1)**	*CjdsbA1*::*cat*	this study
**Δ*dsbA1*Δ*dsbA2 (*KBO2)**	*CjdsbA1*::*kan*, *dsbA2*::*cat*	this study
**Δ*dsbA2 (*AG2)**	*CjdsbA2*::*cat*	[39]
**Δ*dsbB (*AG3)**	*CjdsbB*::*kan*	[39]
**Δ*dsbI (*AG4)**	*CjdsbI*::*cat*	[39]
**Δ*c8j1298* (AB1)**	*c8j_1298*::*kan*	this study
**Δ*dsbD* (AB2)**	*CjdsbD*::*cat*	this study
**Δ*c8j1298*Δ*dsbD* (AB3)**	*c8j_1298*:*kan; CjdsbD*::*spec*	this study
**Δ*dsbA1*Δ1298 (AB4)**	*CjdsbA1*::*cat; c8j_1298*::*kan*	this study
**Δ*dsbA1*/chrA1 [old] (AG1/ chrA1)**	*CjdsbA1*::*kan*/ chr*A1* (chromosomal complementation *CjdsbA1* performed using pUWM1526) Km^R^ Spec^R^	this study
**Δ*dsbA1*/chrA1 [new] (KBO1/chrA1)**	*CjdsbA1*::*cat*/ chr*A1* (chromosomal complementation *CjdsbA1* performed using pUWM1526) Cm^R^ Spec^R^	this study
**Δ*dsbA1*Δ*dsbA2*/chrA2 (KBO2/chrA2)**	*CjdsbA1*::*kan*, *dsbA2*::*cat/* chr*A2* (chromosomal complementation *CjdsbA2* performed using pUWM1516) Km^R^ Cm^R^ Spec^R^	this study
**Δc8j*1298*/chr1298 (AB1/chr1298)**	*c8j_1298*:*kan*/ chr*c8j_1298* (chromosomal complementation *c8j_1298* performed using pUWM1513) Km^R^ Cm^R^	this study
**Δc8j*1298*/pl1298 (AB1/p1501)**	*C8J_1298*:*kan*/ pUWM1501 (plasmid complementation *c8j_1298*) Km^R^ Cm^R^	this study
**Δc8j*1298*Δ*dsbA1*/chrA1 (AB4/chrA1)**	*CjdsbA1*::*cat; c8j_1298*::*kan*/ chr*A1* (chromosomal complementation *CjdsbA1* performed using pUWM1526) Km^R^ Cm^R^ Spec^R^	this study
**Δc8j*1298*Δ*dsbA1*/chr1298 (AB4/chr1298)**	*CjdsbA1*::*cat; c8j_1298*::*kan*/ chr*1298* (chromosomal complementation *c8j_1298* performed using pUWM1517) Km^R^ Cm^R^ Spec^R^	this study

**Table 2:** Plasmids used in this study.

Name	Relevant characteristics	Source/Ref.
**General cloning and expression vectors**		
**pJet 1.2 blunt**	Ap^R^, CloneJET PCR Cloning Kit	ThermoFisher Scientific
**pBluescript KS/SK**	Ap^R^; LacZα	Stratagen
**pET22b**	Ap^R^; *ss’pelB*, IPTG inducible	Novagen
**pET24a**	Km^R^, IPTG inducible	Novagen
**pET28a**	Km^R^, IPTG inducible	Novagen
**pNBSpec**	Spec^R^, source of *spec* cassette	[85]
**pBF14**	Km^R^, source of *aph* cassette	J. van Putten
**pMPMA6**	Ap^R^, Spec^R^, P_araBAD_	[60]
**pRY109**	Cm^R^, source of *cat* cassette	[52]
**pRY111**	Cm^R^, shuttle vector *E*. *coli–C*. *jejuni*	[52]
**pBC49**	Cm^R^, vector for chromosomal complementation vector by double crossing-over in *c8j_0049* pseudogene	this study
**pBS49**	Spec^R^, vector for chromosomal complementation vector by double crossing-over in *c8j_0049* pseudogene	this study
**Plasmid for complementation experiments–final vectors**		
**pUWM1513**	*c8j_1298* in pBC49 –suicide vector for chromosomal complementation in *C*. *jejuni c8j_0049* pseudogene, Cm^R^	this study
**pUWM1517**	*c8j_1298* in pBS49 –suicide vector for chromosomal complementation in *C*. *jejuni c8j_0049* pseudogene, Spec^R^	this study
**pUWM1526**	*CjdsbA1* in pBS49 –vector for chromosomal complementation in *C*. *jejuni c8j_0049* pseudogene, Spec^R^	this study
**pUWM1516**	*CjdsbA2* in pBS49 –suicide vector for chromosomal complementation in *C*. *jejuni c8j_0049* pseudogene, Spec^R^	this study
**pUWM1501**	*c8j_1298* in pRY111 –shuttle vector for plasmid complementation in *C*. *jejuni*, Cm^R^	this study
**pUWM1514**	*ss’pelB-c8j_1298* in pMPMA6 –vector for plasmid complementation in *E*. *coli*, inducible	this study
**Plasmid for complementation experiments–intermediate constructs**		
**pUWM1500**	*c8j_1298* in pJet 1.2 blunt	this study
**pUWM1506**	*CjdsbA1* in pJet 1.2 blunt	this study
**pUWM1505**	*CjdsbA2* in pJet 1.2 blunt	this study
**pUWM1485**	*c8j_1298* in pET22b	this study
**Plasmids for allel-exchange mutagenesis**		
**pUWM1306**	*CjdsbA1*::*aph* in pGEM-T Easy	[39]
**pUWM1458**	*CjdsbA1*::*cat* in pGEM-T Easy	this study
**pUWM1483**	*c8j_1298*::*aph* in pBluescript II KS	this study
**pUWM1503**	*CjDsbD*::*spec* in pBluescript II KS	this study
**pUWM1512**	*CjDsbD*::*cat* in pBluescript II KS	this study
**Plasmids for recombinant Dsb overexpression and purification**		
**pUWM1430**	*CjdsbA1-His*_*6*_ in pET28a	this study
**pUWM1432**	*CjdsbA2-His*_*6*_ in pET24a	this study
**pUWM1462**	*c8j_1298-His*_*6*_ in pET24a	this study
pET28a/*EcdsbA*	*EcdsbA* in pET28a	JFC collection
pET28a/*EcdsbC*	*EcdsbC* in pET28a	JFC collection

### 4.2. DNA manipulation

#### 4.2.1. General DNA manipulations

Standard DNA manipulations were carried out as described in the Sambrook manual [83] or according to manufacturer’s instructions (i.a. A&A Biotechnology, ThermoFisher Scientific). Polymerase chain reactions (PCR) were performed with PrimeStar HS DNA Polymerase (Takara) under standard conditions, according to the manufacturer’s instructions. Synthetic oligonucleotides synthesis and DNA sequencing were performed by Genomed S.A., Warsaw, Poland.

#### 4.2.2. *C*. *jejuni dsb* gene mutagenesis—allele exchange methodology

*C*. *jejuni* 81116 *c8j_1298* and *CjdsbD* mutants were constructed by means of electroporation using suicide plasmids harboring mutated *C*. *jejuni* 81116 DNA. To obtain constructs for *C*. *jejuni c8j_1298/CjdsbD* mutagenesis, both arms of the respective gene were amplified from the *C*. *jejuni* 81116 genome using primer pairs (i.e. 1298_F_Sac– 1298_RXhBXb_2 and 1298_F_XbBXh_2 –c8j_1299_R_Kpn_3; c8j0565_F_Xho–c8j0565_R_NtERIBmC and c8j0565_F_CBmERINt–c8j0565_R_SacI, respectively; S2 Table), designed to create gaps in the *c8j_1298/CjdsbD* nucleotide coding sequences (133 bp and 326 bp, respectively;). PCR products (551 bp and 626 bp, 680 bp and 625 bp, respectively) were directionally cloned into pBluescript, respectively. Subsequently kanamycin/chloramphenicol/spectinomycin resistance cassettes (kanamycin resistance gene excised from pBF14, chloramphenicol resistance gene excised from pRY109 and spectomycin resistance gene from pNBSpec [85]) were inserted between the *c8j_1298/ CjdsbD* gene arms, both in the same transcriptional orientation as inactivated genes. To obtain construct for *dsbA1* mutagenesis, we used pUWM1306 [39]. Using BamHI endonuclease, the kanamycin resistance cassette was replaced by a chloramphenicol resistance cassette to form pUWM1458. Correct construction of pUWM1483 (*c8j_1298* with kanamycin resistance cassette), pUWM1458 (*CjdsbA1* with chloramphenicol resistance casette), pUWM1512 (*CjdsbD* with a chloramphenicol resistance cassette) and pUWM1503 (*CjdsbD* with a spectomycin resistance cassette) was confirmed by sequencing. Recombinant plasmids pUWM1458, pUWM1483 and pUWM1512 were electrotransformed into *C*. *jejuni* 81116 WT competent cells. Double cross-over of the three single mutants (KBO1, AB1 and AB2, respectively) was verified by PCR analysis and sequencing. For Δ*c8j_1298* mutant strain we verified if the introduced mutation did not affect the following *c8j_1299* gene transcription. Next, to construct the double mutant strains Δ*c8j_1298*Δ*CjdsbD* (AB3) and Δ*CjdsbA1*Δ*c8j_1298* (AB4), pUWM1503 or pUWM1458 was electrotransformed into *C*. *jejuni* 81116 Δ*c8j_1298* (AB1) competent cells. To generate double mutant Δ*CjdsbA1*Δ*dsbA2* (KBO2) pUWM1306 was introduced to *CjdsbA2* deficient strain (AG2).

#### 4.2.3. Construction of vectors for complementation experiments

*Construction of c8j_1298 plasmids for complementation experiments in C*. *jejuni*. To analyze the complementation of the Δ*c8j_1298* mutation in *C*. *jejuni* 81116 by a wild type copy of C8J_1298, recombinant plasmid was constructed based on shuttle *E*. *coli/C*. *jejuni* plasmid: pRY111 [52]. The Δ*c8j_1298* coding nucleotide sequence with its own promoter was amplified from *C*. *jejuni* 81116 genomic DNA, using a pair of primers: C8J_1298_SpeIBamHI_Fk–C8J_1298XhoI_Rk. The purified PCR products, as well as the shuttle plasmid, were digested with XhoI/BamHI and ligated. Correct construction of pUWM1501 was confirmed by sequencing. Obtained vector was introduced into *C*. *jejuni* 81116 strains (Table 1).

*Construction of C8J_1298*^*+*^ *plasmids for in trans complementation experiments in E*. *coli*. To perform complementation experiments in *E*. *coli* cells, *c8j_1298* was cloned with the *E*. *coli pelB* signal sequence (*ss’pelB*) under an inducible arabinose promoter. Nucleotide sequence of *c8j_1298* (without promoter and signal sequence) was amplified from *C*. *jejuni* 81116 genomic DNA, using a pair of primers: C8J_1298_BamHI—C8J_1298_Xho_stop. The purified PCR product and pET22b were digested with BamHI/XhoI and ligated together, forming pUWM1485. The *c8j_1298* gene with *ss’pelB* was excised from pUWM1485 with NdeI/Klenow fragment and subsequently with XhoI endonuclease, then cloned into pMPM-A6 [60] digested with EcoRI/Klenow fragment and subsequently with XhoI endonuclease. Correctness of the obtained pUWM1514 was confirmed by sequencing and restriction analysis, and the plasmid was then transformed into *E*. *coli* lacking *dsbA/ dsbAdsbB/ dsbC* genes (Table 1).

*Construction of plasmids for chromosomal complementation experiments*. To obtain constructs for chromosomal complementation by selected gene introduction into *C*. *jejuni c8j_0049* pseudogene, both arms of the pseudogene were amplified from the *C*. *jejuni* 81116 genome using primer pairs (c8j0048Sac–c8j0049Not and c8j0049Xho–c8j0050Kpn; S2 Table) designed to create a gap in the pseudogene nucleotide sequence (933 bp). PCR products (1328 bp and 1412 bp) were directionally cloned into pBluescript KSII. Subsequently, chloramphenicol and spectinomycin resistance cassettes (*cat* gene excised from pRY109, spectomycin resistance gene from pNBSpec) were inserted between the pseudogene arms, in an orientation opposite to the inactivated genes. In the space between the arms and the cassette a few restriction enzyme sites were left to allow cloning genes of interest under their own promoter sequence. Correct construction of novel chromosome complementation plasmids pBC49 and pBS49 was confirmed by sequencing. To confirm correctness of constructed plasmids, control electroporation to *C*. *jejuni* 81116 was performed. Introduction of the appropriate DNA fragment by double crossing-over into the sequence of pseudogene *c8j_0049* was verified by PCR analysis and sequencing. Next, the nucleotide coding sequence of *c8j_1298*/*CjdsbA1*/*CjdsbA2* with its own promoter was amplified from the *C*. *jejuni* 81116 genome using primer pairs C8J_1298_SpeIBamHI_Fk–C8J_1298XhoI_Rk; C8J0813_Spe–C8J0813_Xho; C8J0811_Spe–C8J0811_Xho (S2 Table) and cloned into pJet 1.2/blunt vector to form pUWM1500, pUWM1506 and pUWM1505, respectively. Using XbaI and NotI restriction enzymes, fragments coding *c8j_1298* or *CjdsbA1/CjdsbA2* with their own promoter sequences were cloned into pBC49 and pBS49 respectively, in the identical transcriptional orientation as the cassettes. Correct construction of plasmids pUWM1513, pUWM1517 pUWM1526 and pUWM1516 was confirmed by sequencing. Subsequently, the plasmids were introduced by electroporation into appropriate *C*. *jejuni* 81116 strains. Double cross-over in the sequence of pseudogene *c8j_0049* was verified by PCR analysis and sequencing. Resulting strains were used for the complementation assays.

### 4.3. Protein analysis and biochemical assays

#### 4.3.1. Overexpression and purification of proteins for biochemical experiments and rabbit immunization

All the proteins used for biochemical experiments were overexpressed from *E*. *coli* BL21 or Rosetta strains harboring the appropriate plasmids (Tables 1 and 2) by autoinduction [86]. The expression vector carrying *c8j_1298*, *CjdsbA1* or *CjsbA2* was constructed by amplifying the region encoding the mature protein with primers: C8J_1298_Nde–C8J_1298_Xho, C8J814_Nco–C8J814_Xho, C8J811_Nde–C8J811_Xho, respectively. Next the DNA fragments were inserted into pET24a (*c8j_1298* and *CjdsbA2*) using NdeI and XhoI restriction enzymes or into pET28a (*CjdsbA1*) using NcoI and XhoI restriction enzymes. For biochemical assays, proteins were expressed and purified from *E*. *coli* Rosetta. EcDsbA and/or EcDsbC *E*. *coli* proteins were overexpressed from *E*. *coli* BL21 harboring pET28a/EcDsbA, EcDsbC (Table 1). After induction, cultures were centrifuged (4000 g) and the cell pellet was suspended in 50 mM sodium phosphate, pH 8.0, 300 mM NaCl, 10 mM imidazole. Cells were disrupted by ultrasonication. The cell lysate was centrifuged (8000 g) and the resulting supernatant was applied onto Bio-Scale Mini Profinity IMAC® Cartridges (Bio-Rad) containing Ni-charged resin. The protein was eluted with an imidazole gradient, using the NGC chromatography system (Bio-Rad). To obtain higher purity, proteins were next loaded onto ENrich®SEC 70 size exclusion columns (Bio-Rad) and eluted with PBS. For biochemical tests, samples were dialyzed on desalting columns (Bio-Rad) against phosphate buffer, pH 7.0 (insulin test) or PBS buffer (oxidative and refolding tests of RNaseA) and concentrated as needed (Amicon® Ultra-4, 10,000 NMWL; Millipore). C8J_1298, DsbA1, DsbA2 were used for rabbit immunization (Animal Facility, Faculty of Biology and Environment Protection, University of Lodz; Ethics committee agreement number 280/2017 (date 22.02.2017) and 746/2015 (date 21.05.2015) I Local Ethics Committee for animal experiments in Warsaw).

#### 4.3.2. Determination of the *in vivo* redox state of proteins

The redox states of the analyzed *C*. *jejuni* proteins were visualized by alkylating the free cysteine residues using 4-acetamido-4’-maleimidylstilbene-2,2’-disulfonic acid (AMS, ThermoFisher Scientific) [1, 47]. These agents can only modify covalently free thiols, resulting in a molecular mass increase of 490 Da (AMS). Briefly, *C*. *jejuni* cells were harvested from BHI-SG liquid cultures after 16–18 h of incubation under microaerobic conditions. *E*. *coli* cells were grown in liquid culture in LB broth until the OD600 nm value was 0.6 in standard conditions. Samples were centrifuged and pellets were resuspended in sterile PBS. Next, ice-cold trichloroacetic acid (TCA, final concentration 10% v/v) was immediately added directly to the culture. Whole-cell proteins were precipitated and collected by centrifugation, washed with ice-cold acetone, and then dissolved in 50 mM Tris-HCl (pH 7.5), 10 mM EDTA, 0.1% (v/v) SDS containing 20 mM AMS by agitation (1400 rpm) for 45 min at 37°C. The proteins in non-reducing Laemmli buffer were resolved by 14% SDS-PAGE without reducing agent. Proteins were then detected by Western-blot analysis using a specific serum. As controls, we used samples previously treated with 100 mM DTT for 30 min at 30°C before precipitation of the proteins with TCA. Fractions of reduced and oxidized protein were determined using Image Lab^™^ software (Bio-Rad). Result is the average of three independent experiments, with two technical repetitions (n = 3).

#### 4.3.3. Insulin reduction assay

Reductase activity was assessed by an insulin precipitation assay [3, 84] using human insulin solution (Sigma) Reactions (triplicate) were carried out in 200 μl of 100 mM sodium phosphate buffer, pH 7.0, 133 μM insulin, 1 mM dithiothreitol (DTT), 2 mM EDTA and 10 μM of C8J_1298 or EcDsbA or EcDsbC; reaction mixtures were incubated in a 96-well plate format at room temperature in a Sunrise^™^ (Tecan) plate reader [47]. Reactions were started by adding DTT to a final concentration of 1 mM. The changes in absorbance (A650nm) as a function of time were measured [3, 87]. Result is the average of three independent experiments, with two technical repetitions (n = 3).

#### 4.3.4. Determination of the redox potential of C8J_1298 protein

The amount of C8J_1298 present as reduced and oxidized forms was determined using 4-acetamido-4′-maleimidylstilbene-2,2′-disulfonic acid (AMS) trapping [51]. Briefly, C8J_1298 (2 μM) was incubated overnight at room temperature in 50 mM KPi pH 7.0, 0.1 mM EDTA and various glutathione (GSH)/glutathione disulfide (GSSG) ratios. After incubation, proteins were precipitated with trichloroacetic acid (TCA) (10% final concentration). After 20-min incubation on ice, the samples were centrifuged (20000 g, 5 min, 4°C), and the pellets were washed with cold acetone. After a second centrifugation, pellets were dried and resuspended in a buffer containing 20 mM AMS, 0.1% SDS, 10 mM EDTA, and 50 mM Tris-HCl (pH 7.5). After 45-min incubation at 37°C, with 1,400 rpm shaking, samples were loaded onto 14% SDS-polyacrylamide gels under denaturing conditions. Fractions of reduced (band intensity) protein (R) were determined using Image-Lab. The redox potential was calculated as described previously [70, 88]. First, the equilibrium constant (K_eq_) was calculated from the equation:

$$R=\left({\left[GSH\right]}^{2}/\left[GSSG\right]\right)/\left(K_{eq}+{\left[GSH\right]}_{2}/\left[GSSG\right]\right)$$

The standard redox potential was calculated from the Nernst equation using the glutathione standard potential:

E°'C8J_1298=E°'GSH/GSGG(RT/2F)lnKeq

E°^'GSH/GSGG^–glutathione standard potential -240 mV

R–gas constant (8.314 J K^-1^ mol^-1^)

T–temperature (K)

F–Faradaya constant 96485 C mol^-1^

K_eq_−equilibrium constant

Result is the average of three independent experiments, with two technical repetitions (n = 3).

#### 4.3.5. Oxidative folding of reduced RNaseA

*In vitro* oxidative folding of reduced, unfolded RNaseA (ruRNaseA) was performed with C8J_1298, EcDsbC and EcDsbA as described earlier [47]. Proteins were oxidized with 50 mM oxidized glutathione (GSSG) and incubated for 1 h at room temperature. RNaseA was reduced by overnight incubation at room temperature in 100 mM Tris acetate pH 8.0, containing 6 M guanidine hydrochloride and 140 mM DTT. All proteins were then dialyzed on desalting columns (Bio-Rad) and concentrated in PBS. Native RNaseA and EcDsbA were used as positive controls. The redox state of the thiols was confirmed by the Ellman’s assay, which exploits the colorimetric change at A412nm when 5,5'-dithiobis-(2-nitrobenzoic acid) (DTNB; ThermoFisher Scientific) is converted to 2-nitro-5 thiobenzoate upon cleavage of the disulfide bond by free thiols. Oxidase activity was measured by analyzing the cleavage of cCMP (Sigma; cytidine 2′:3′-cyclic monophosphate monosodium salt) at A296 nm by refolded RNaseA in the presence of tested enzymes. Reactions (triplicate) were carried out in 200 μl of PBS buffer containing 100 mMTris acetate pH 8.0, 2 mM EDTA, 0.2 mM GSSG, 1 mM GSH, 4.5 mM cCMP, RNaseA (10 μM) and analyzed enzyme (20 μM). The reaction mixtures were prepared in a 96-well plate format and read through 30–60 min at 27°C in a Sunrise^™^ (Tecan) plate reader. Three independent experiments were performed.

#### 4.3.6. Refolding of scrambled RNaseA (scRNase)

*In vitro* refolding of scrambled RNaseA was performed for C8J_1298 and EcDsbC as described earlier [47]. Proteins were reduced with 100 mM DTT and incubated overnight at 4°C. RNaseA was first reduced by overnight incubation at room temperature in 100 mM Tris acetate pH 8.0, containing 6 M guanidine hydrochloride and 140 mM DTT. Then, in order to introduce incorrect disulfides, the reduced RNaseA was dialyzed against PBS buffer containing 6 M guanidine hydrochloride, sparged with oxygen and incubated for 3 days in the dark at room temperature. Finally, 2 mM hydrogen peroxide (Sigma) was added for 30 min at 25°C. All proteins were then dialyzed on desalting columns (Bio-Rad) and concentrated in PBS. EcDsbC was used as a positive control. The redox state of the thiols was confirmed by the Ellman’s assay. RNaseA activity was measured by analyzing the cleavage of cCMP, as described above for the oxidative test, but with the reaction mixture changed to 100 mM Tris acetate pH 8.0, 2 mM EDTA, 10 μM DTT, 4.5 mM cCMP, RNaseA (40 μM) and analyzed enzyme (20 μM). Three independent experiments were performed.

#### 4.3.7. Determination of the oligomeric state of C8J_1298 using glutaraldehyde

Crosslinking of polypeptide chains with glutaraldehyde was performed essentially as described [70, 89]. C8J_1298 (1.8 mg/ml) was incubated at room temperature with different concentrations of glutaraldehyde (0.0001–0.02% v/v) in 0.05 M bicine–NaOH buffer (pH 8.5), 0.1 mM DTT, 0.4 M NaCl for 20 min and the reaction was quenched by adding ethanolamine–HCl (pH 8.0) to a final concentration of 0.14 M [70]. Three independent experiments were performed.

### 4.4. Phenotype assays

#### 4.4.1. Spot titers for cadmium, DTT and copper resistance

Spot titers for cadmium, copper and DTT resistance were performed to evaluate the oxidase or isomerase activity of *C*. *jejuni* mutants *in vivo* [49]. Briefly, *C*. *jejuni* cells were harvested from BA plates after 16–18 h of incubation under microaerobic conditions. Samples were standardized using OD600 nm of the culture and serially diluted with LB medium. Then, 3 μl of each dilution was plated onto BA plates, supplemented with 20 μM cadmium chloride, 0.8 mM copper sulfate or 8 mM DTT. *C*. *jejuni* cells were incubated 48 hours under microaerobic conditions. All spot titers were performed in triplicate. *E*. *coli* cells were grown in liquid culture in LB broth until the OD600 nm value was 0.6 and serially diluted (10-fold) with 150 mM NaCl. Then, 3 μl of each dilution was plated onto BHI media supplemented with arabinose and 4 mM or 8 mM CuCl_2_. Strains were grown at 34°C. All spot titers assays were performed in triplicate (n = 3).

#### 4.4.2. Motility assay

Motility assays were performed as described earlier [39]. *C*. *jejuni* cells were harvested from BA plates after 16–18 h of incubation under microaerobic conditions. Samples were standardized using OD600 nm of the culture. Next, 4 μl of bacterial culture was spotted onto Mueller-Hinton (MH) soft agar plates containing 0.35% (w/v) agar, and incubated for 48 hours at 37°C under microaerobic conditions. *E*. *coli* cells were grown in liquid culture in LB broth supplemented with arabinose until the OD600 nm value was 0.6. Then, 4 μl of bacterial culture was spotted onto LB soft agar plates containing 0.35% (w/v) agar, 0.2% (v/v) arabinose and incubated overnight at 37°C. All motility assays were performed in triplicate (n = 3).

#### 4.4.3. Mucoid phenotype assay

The *E*. *coli* Δ*mdoG*Δ*dsbC*::*kan* strain harboring various recombinant plasmids (pBAD33, pBAD33/DsbC^+^, pMPMA6/C8J_1298^+^) were grown on M63 minimal medium supplemented with arabinose for 2 days at room temperature. The mucoid phenotype of the analyzed strains was evaluated. The assay was performed in triplicate (n = 3).

#### 4.4.4. Oxidative stress

To examine susceptibility to oxidative stress, we performed viability assays as previously described [58, 90, 91]. Briefly, *C*. *jejuni* cells were harvested from BHI-SG liquid cultures (16–18 h of incubation under microaerobic conditions) and OD600 nm was adjusted to 0.5. Then bacterial cells were exposed to H_2_O_2_ at final concentrations of 10 or 15mM for 60 min, all at 37°C under microaerobic conditions. Serial dilutions (10-fold) were prepared with BHI medium. Then, 3 μl of each dilution was plated onto BA plates and incubated under microaerobic conditions for 48 h. The assay was performed in triplicate (n = 3).

#### 4.4.5. Assesment of AstA activity

Qualitative assays for AstA (arylsulfatase) activity were carried out as described previously [39, 92]. Briefly, *C*. *jejuni* was grown for 16 hours on MH plates supplemented with XS (5-bromo-4-chloro-3-indolylsulfate, 100 mg/ml), a substrate for arylsulfatase, and bacterial colonies were observed for blue color acquisition, which indicates AstA activity. The assay was performed in triplicate (n = 3).

### 4.5. RNA manipulations

#### 4.5.1. RNA isolation

*C*. *jejuni* strains were grown on BA plates for 16–18 h as described above. Cells were then harvested from one plate and total RNA was isolated using the Total RNA Mini (A&A Biotechnology), according to the manufacturer’s recommendations. Contaminating DNA was removed using TURBO DNA-free^™^ Kit (Invitrogen–ThermoFisher Scientific), according to the manufacturer’s instructions. RNA concentration and RNA quality were measured using Nano Drop 2000 (ThermoFisher Scientific) and 2100 Bioanalyzer (AgilentTechnologies) [93].

#### 4.5.2. REAL-TIME qRT-PCR

cDNAs were obtained by reverse transcription of 10μg of total RNA using the Maxima First Strand cDNA Synthesis Kit for qPCR-RT, according to the manufacturer’s instruction (ThermoFisher Scientific). Real-time PCR using 5×HOTFIREPol® EvaGreen® qPCR Mix Plus (ROX) (SolisBioDyne) was carried out on a LightCycler 96 (Roche). HPLC purified oligonucleotide primer pairs specific for each gene of interest (S2 Table) were purchased from Genomed. Relative quantification of gene transcription was performed using the comparative Ct (threshold cycle) method. The relative amount of target cDNA was normalized using the *gyrA* gene as an internal reference standard [44]. Result is the average of three independent experiments, with two technical repetitions (n = 3) [93].

### 4.6. Bioinformatics analysis

Protein sequences of *C*. *jejuni* 81116 containing one or more cysteine residues were analyzed with SignalP [94] and TMHMM [95] in order to detect signal peptides and transmembrane helices, respectively. Note that cysteine residues localized within signal peptides were not considered in the counting.

BLAST [96] searches against NCBI RefSeq database [97] were initialized with catalytic domains of C8J_1298, HP0231, and *Escherichia coli* DsbG and DsbC proteins. Hits with e-value >1e-3 or query coverage <90% were discarded resulting in a non-redundant set of 1610 sequences. These sequences were clustered using CLANS [98], and groups corresponding to DsbG-like, DsbC-like and C8J_1298/ HP0231-like families were defined manually at 1e-14 BLAST cut-off. Sequences in each group were aligned using MUSCLE [99] and used to calculate sequence logos with WebLogo [100]. From each group, a set of representative sequences was manually selected. Moreover, we extracted other related Dsb catalytic domain sequences defined in our previous study [47]. All the sequences were aligned using MUSCLE [99] and used to build a phylogenetic tree with FastTree [101]. The additional related Dsb sequences served as an outgroup and were used to root the tree. The tree was visualized using ETE3 toolkit [102] (for clarity, the outgroup sequences are omitted in Fig 1).

## Supporting information

S1 Table List of potential *dsb* genes in C. jejuni 81116 and orthologs in other strains. (DOCX)

S2 Table Primers used in this study. (DOCX)

S1 Fig Cysteine distribution in C. jejuni 81116 proteins. (TIFF)

S2 Fig The redox equilibrium C8J_1298 with glutathione corresponds to that determined for EcDsbA. The fraction of reduced (R) C8J_1298 was determined in various glutathione (GSH)/glutathione disulfide (GSSG) ratios using AMS reagent. Fractions (band intensity) of reduced C8J_1298 were determined using Image-Lab (BIO-RAD) after resolving on 14% SDS-PAGE. The standard redox potential was calculated from the Nernst equation using the glutathione standard potential. (A) The bars represent the average of three independent experiments, with two technical repetitions (n = 3). (B) The result of one representative experiment.
(TIF)

S3 Fig Western blot analysis confirming presence of absence of (A) C8J_1298 and (B) CjDsbA1 in various *C*. *jejuni* 81116 strains. Complementation of the Δ*c8j_1298* mutation restores the presence of C8J_1298. Plasmid complementation (Δ*c8j_1298*/pl1298) of the Δ*c8j_1298* mutation abolishes production of CjDsbA1. *C*. *jejuni* 81116 strains proteins (the whole cell lysate) were separated by 12% SDS-PAGE and electrotransferred onto a nitrocellulose membrane. Specific rabbit sera with antibodies against **(A)** C8J_1298 or **(B)** CjDsbA1 were used to verify the absence or presence C8J_1298/CjDsbA1 in *C*. *jejuni* 81116 cells.
(TIF)

S4 Fig *c8j_1299* expression in *C*. *jejuni* 81116 wild type cells and in Δ*c8j_1298* (AB1) strain. To verify if *c8j_1299* transcription is affected by mutation of *c8j_1298*, total RNA of *C*. *jejuni* strains was isolated, contaminating DNA was removed and cDNA was obtained by reverse transcription. *C*. *jejuni* 81116 and Δ*c8j_1298* cDNA was amplified in a standard PCR reaction with primer pair c8j1299-RT–c8j1299-RT2. As a control, *C*. *jejuni* 81116 genomic DNA was used. *c8j_1299* transcript was present in wild type as well as in Δ*c8j_1298* mutant cells. (*) unspecific band.
(TIF)

S5 Fig Plasmid complementation of Δ*c8j_1298* mutation results in *dsbA1* transcription level decrease. mRNA was isolated from *C*. *jejuni* 81116 strains. Results from *dsbA1* amplification were normalized using *gyrA* expression and presented as fold decrease *dsbA1*. Data presented is the average of three independent experiments, with two technical repetitions (n = 3). p>0,001 ***; p>0,005 **; ns—not significant.
(TIF)

S6 Fig *C*. *jejuni c8j_1298* gene is expressed in *E*. *coli* strains from induced arabinose promoter. *E*. *coli* strains harboring recombinant plasmids were cultured with (+) or without (-) arabinose induction. The *c8j_1298* gene was cloned into pMPM-A6 under control of arabinose induced promoter. Proteins (whole cell lysates) from analyzed strains were separated by 12% SDS-PAGE, electrotransferred onto a nitrocellulose membrane and developed with rabbit anti-C8J_1298 serum.
(TIF)

S1 File Multiple sequence alignment of Dsb catalytic domains in FASTA format. The alignment contains 91 sequences shown in Fig 1 and 43 outgroup sequences used to root the tree.
(FASTA)

S2 File The unrooted phylogenetic tree in Newick format calculated based on the multiple sequence alignment from S1 File. (NWK)

S3 File A rooted variant of the phylogenetic tree from S2 File in Newick format. After rooting, leafs corresponding to the outgroup sequences were removed.
(NWK)

S1 Text Verification of the CjDsbA1 (AG1 and KBO1) mutant. (DOCX)

S1 Raw images (PDF)
