# Supplementary material for: C8J_1298, a bifunctional thiol oxidoreductase of Campylobacter jejuni, affects Dsb (disulfide bond) network functioning
Source: PLoS One. 2020 Mar 23;15(3):e0230366. doi: 10.1371/journal.pone.0230366 (PMC7089426; doi:10.1371/journal.pone.0230366)
Supplement: S1 Table — (DOCX) [file pone.0230366.s001.docx]

### S1 Table. List of potential *dsb* genes in *C. jejuni* 81116 and orthologues in other strains

| Gene in  *C. jejuni* 81116 | Corresponding gene in  *C. jejuni*  81-176 | Corresponding gene in  *C. jejuni* 11168 | CXXC motif | Designation | Protein localization and other comments | References |
| --- | --- | --- | --- | --- | --- | --- |
| ***c8j_0814*** | *cjj81176_0883* | *cj0872* | CIHC | DsbA1 | Periplasm | [2, 39] |
| ***c8j_0811*** | *cjj81176_0880* | pseudogene | CTHC | DsbA2 | Periplasm | [2, 39] |
| ***c8j_0812*** | *cjj81176_0881* | *cj0865* | CEQC | DsbB | Inner membrane | [39, 59] |
| ***c8j_0016*** | *cjj81176_0044* | *cj0017c* | CTLC | DsbI | atypical DsbB | [39, 59] |
| ***c8j_1298*** | *cjj81176_1382* | *cj1380* | CPYC | DsbC | Periplasm  Bifunctional thiol oxidoreductase | This work |
| ***c8j_0565*** | *cjj81176_0631* | *cj0603c* | CENC | DsbD | Inner membrane  Cytochrome c biogenesis  C8J_1298 redox partner | [40]  This work |
| ***c8j_1047*** | *cjj81176_1124* | *cj1106* | CTPC | CcmG | Anchored in inner membrane  Cytochrome c biogenesis  Lipoprotein | [40] |
| ***c8j_1150*** | *cjj81176_1222* | *cj1207c* | CTPC | CcmG | Anchored in inner membrane  Cytochrome c biogenesis  Lipoprotein | [40] |
| ***c8j_1566*** | *cjj81176_1655* | *cj1664* | CPSC | ------------ | Unknown function  Lipoprotein  Iron regulated | [40, 42]  This work |
| ***c8j_1567*** | *cjj81176_1656* (shorter form) | *cj1665* | CGVC | ------------ | Lipoprotein  Unknown function  Iron regulated | [40, 42] |
| ***c8j_1568*** | *cjj81176_1657* | *cj1666c* | CGCC | ------------ | Periplasm  Unknown function  Iron regulated | [42]  This work |
| ***c8j_0031*** | *cjj81176_0065* | Not present | CVYC | ------------ | Biogenesis of  sulphate reductase  Small thioredoxin  Lipoprotein | [40]  This work |

References

2. Bocian-Ostrzycka, K. M.; Grzeszczuk, M. J.; Dziewit, L.; Jagusztyn-Krynicka, E. K., Diversity of the *Epsilonproteobacteria* Dsb (disulfide bond) systems. *Front Microbiol* **2015,** *6*, 570. [10.3389/fmicb.2015.00570].

39. Grabowska, A. D.; Wywial, E.; Dunin-Horkawicz, S.; Lasica, A. M.; Wosten, M. M.; Nagy-Staron, A.; Godlewska, R.; Bocian-Ostrzycka, K.; Pienkowska, K.; Laniewski, P.; Bujnicki, J. M.; van Putten, J. P.; Jagusztyn-Krynicka, E. K., Functional and bioinformatics analysis of two *Campylobacter jejuni* homologs of the thiol-disulfide oxidoreductase, DsbA. *PLoS One* **2014,** *9*, e106247. [10.1371/journal.pone.0106247].

40. Liu, Y. W.; Kelly, D. J., Cytochrome c biogenesis in Campylobacter jejuni requires cytochrome c6 (CccA; Cj1153) to maintain apocytochrome cysteine thiols in a reduced state for haem attachment. *Mol Microbiol* **2015,** *96*, 1298-317. [10.1111/mmi.13008].

42. Holmes, K.; Mulholland, F.; Pearson, B. M.; Pin, C.; McNicholl-Kennedy, J.; Ketley, J. M.; Wells, J. M., Campylobacter jejuni gene expression in response to iron limitation and the role of Fur. *Microbiology* **2005,** *151*, 243-57. [10.1099/mic.0.27412-0].

44. Svensson, S. L.; Huynh, S.; Parker, C. T.; Gaynor, E. C., The Campylobacter jejuni CprRS two-component regulatory system regulates aspects of the cell envelope. *Mol Microbiol* **2015,** *96*, 189-209. [10.1111/mmi.12927].

59. Raczko, A. M.; Bujnicki, J. M.; Pawlowski, M.; Godlewska, R.; Lewandowska, M.; Jagusztyn-Krynicka, E. K., Characterization of new DsbB-like thiol-oxidoreductases of Campylobacter jejuni and Helicobacter pylori and classification of the DsbB family based on phylogenomic, structural and functional criteria. *Microbiology* **2005,** *151*, 219-31. [10.1099/mic.0.27483-0].
