# Supplementary material for: C8J_1298, a bifunctional thiol oxidoreductase of Campylobacter jejuni, affects Dsb (disulfide bond) network functioning
Source: PLoS One. 2020 Mar 23;15(3):e0230366. doi: 10.1371/journal.pone.0230366 (PMC7089426; doi:10.1371/journal.pone.0230366)
Supplement: S2 Table — (DOCX) [file pone.0230366.s002.docx]

**S2 Table. Primers used in this study**

| Name | Sequence | Restriction Site | Source |
| --- | --- | --- | --- |
| Primers for mutagenesis | | | |
| 1298_F_Sac | GAGCTCTTAATTCTTGCCTGTTCAGC | SacI | This study |
| 1298_RXhBXb_2 | CTCGAGGGATCCTCTAGAGCTTCTTTGTAGATTAGAGCGG | XbaI, BamHI, XhoI | This study |
| 1298_F_XbBXh_2 | TCTAGAGGATCCCTCGAGTTAGGTCTTAGCGCTACTCC | XbaI, BamHI, XhoI | This study |
| c8j_1299_R_Kpn_3 | GGTACCAAGAGTTTTTATACTCTTGATTGTC | KpnI | This study |
| c8j0565_F_Xho | GACCTCGAGTTGCTTCTATATTGTCTTTAAATG | XhoI | This study |
| c8j0565_R_NtERIBmC | GAGGCGGCCGCGAATTCGGATCCATCGATGAGTAGCATAGGCTAAAGAC | ClaI, NotI, EcoRI, BamHI | This study |
| c8j0565_F_CBmERINt | CTCATCGATGGATCCGAATTCGCGGCCGCCTCTTGGCATGGGTATACCTTTG | ClaI, NotI, EcoRI, BamHI | This study |
| c8j0565_R_SacI | GACGAGCTCTTCTTTCATGGTTTTAATTTG | SacI | This study |
| Primers for verifying | | | |
| Cjj882_3 | AGCGCAACAGCTGGAATG GC | ø | [39] |
| Cjj884_1 | TGCCTCAAGGTGCGCCTG AC | ø | [39] |
| c8j1299-RT | GACAGCTTTTTTATAGCACTC | ø | This study |
| c8j1299-RT2 | GACATAATTGACAGCGTTCT | ø | This study |
| c8j46-1a | GTGTGC CGCAGT TATCTG GG | ø | This study |
| c8j46-2 | CGAAATGGAAGCAAAGAAATAGT | ø | This study |
| Primers for complementation | | | |
| c8j0048_Sac | GCCGAGCTCTGTAGTGCATCTTC | SacI | This study |
| c8j0049_Not | CAGGCGGCCGCAGTGGTAATGCCACTAAG | NotI | This study |
| c8j0049_Xho | CTTCTCGAGTCTGCTATGGGACTTG | XhoI | This study |
| c8j0050_Kpn | GTTGGTACCTTCTAGTCGCTATGAG | KpnI | This study |
| C8J_1298_SpeIBamHI_Fk | GACACTAGTGGATCCTGCCATGCTTTCAG | SpeI, BamHI | This study |
| C8J_1298XhoI_Rk | GACCGTCTCGAGGCCTTCATTGCTTGTTTG | XhoI | This study |
| C8J_1298_BamHI | GTCGGATCCGGCAAGTAATTCAGAAATTAGTG | BamHI | This study |
| C8J_1298_Xho_stop | CGCTCGAGTTTATTTAATAATAGTTGGAGTAG | XhoI | This study |
| C8J0813_Spe | GAACTAGTTCTCTAACTCAATATGAACCAGATA | SpeI | This study |
| C8J0813_Xho | CTACTCGAGGTAGGACCTTATGATGATAG | XhoI | This study |
| C8J0811_Spe | GTTACTAGTCTAAATCTAAAATTTTTAGCTTTTTAAC | SpeI | This study |
| C8J0811_Xho | GGTCTCGAGCATTGTTCACAAGGTTCCATAA | XhoI | This study |
| Primers for recombinant proteins | | | |
| C8J_1298_Nde | GCACATATGGCAAGTAATTCAGAAATTAG | NdeI | This study |
| C8J_1298_Xho | CGACCGAGTTTAATAATAGTTGGAGTAGC | XhoI | This study |
| C8J814_Nco | CAGCCA TGGCGA ATAGTT TTATTA CCCTTA ATC | NcoI | This study |
| C8J814_Xho | CAGCTC GAGTTT CATATT GCTTAA TTTTTT AAC | XhoI | This study |
| C8J811_Nde | GCACAT ATGGAA GGTAAA GAATAT ATAATT C | NdeI | This study |
| C8J811_Xho | GCACTC GAGTTT TTGTTT GCTAAG TTCTTT AG | XhoI | This study |
| Primers for qRT-PCR | | | |
| q_C8JDsbA1_s | CTATCCTGTAAGTTTAATGAATGG | ø | This study |
| q_C8JDsbA1_as | CTATCAGAATAACTCGCATCTT | ø | This study |
| q_gyrA_s | CTTTGCCTGACGCAAGAGATGGTT | ø | [44] |
| q_gyrA_as | AGCACCCACTATACGGGCTGATTT | ø | [44] |
