# Supplementary figures and images for: C8J_1298, a bifunctional thiol oxidoreductase of Campylobacter jejuni, affects Dsb (disulfide bond) network functioning
Source: PLoS One. 2020 Mar 23;15(3):e0230366. doi: 10.1371/journal.pone.0230366 (PMC7089426; doi:10.1371/journal.pone.0230366)

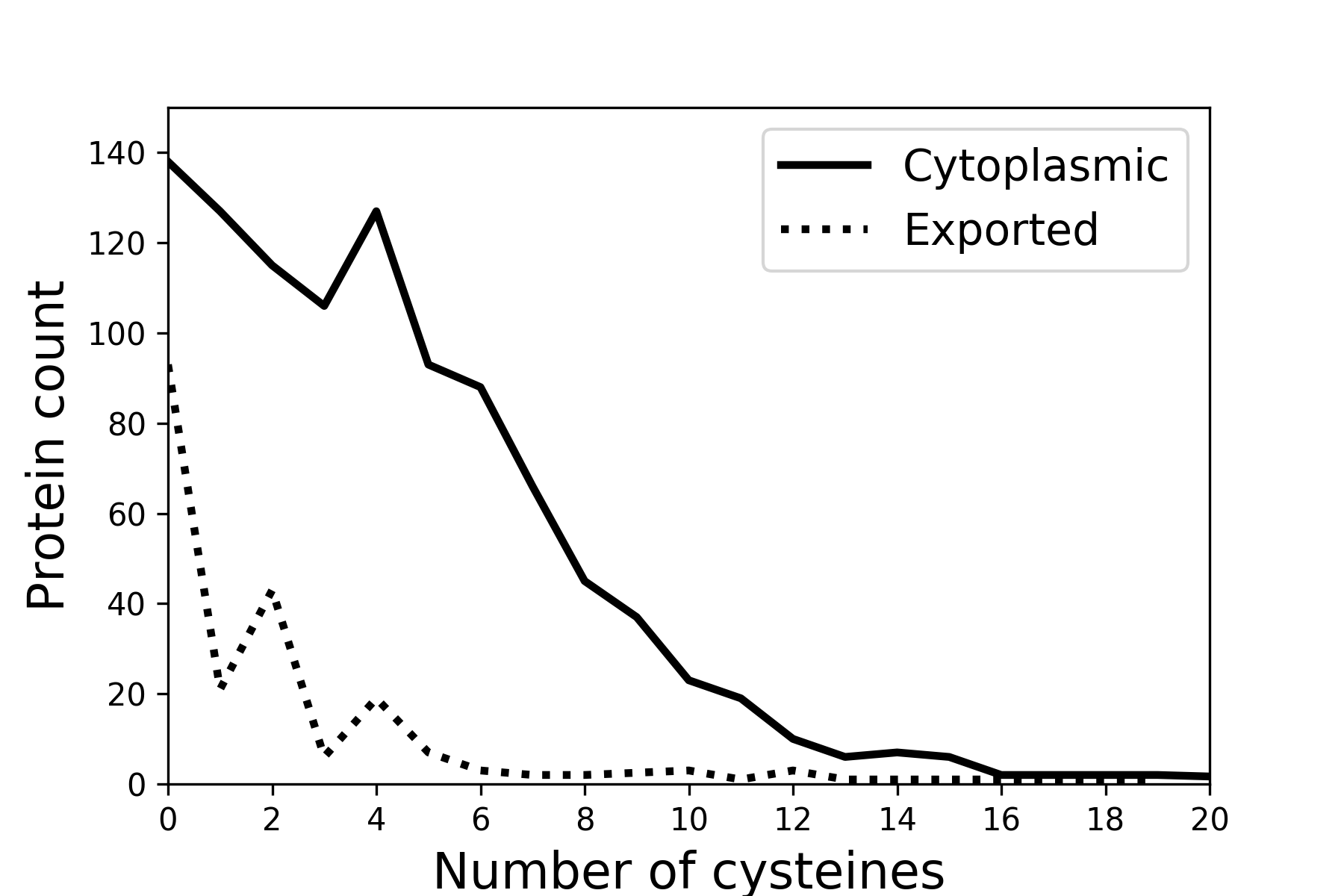

Supplement: S1 Fig — (TIFF) [file pone.0230366.s003.tiff]

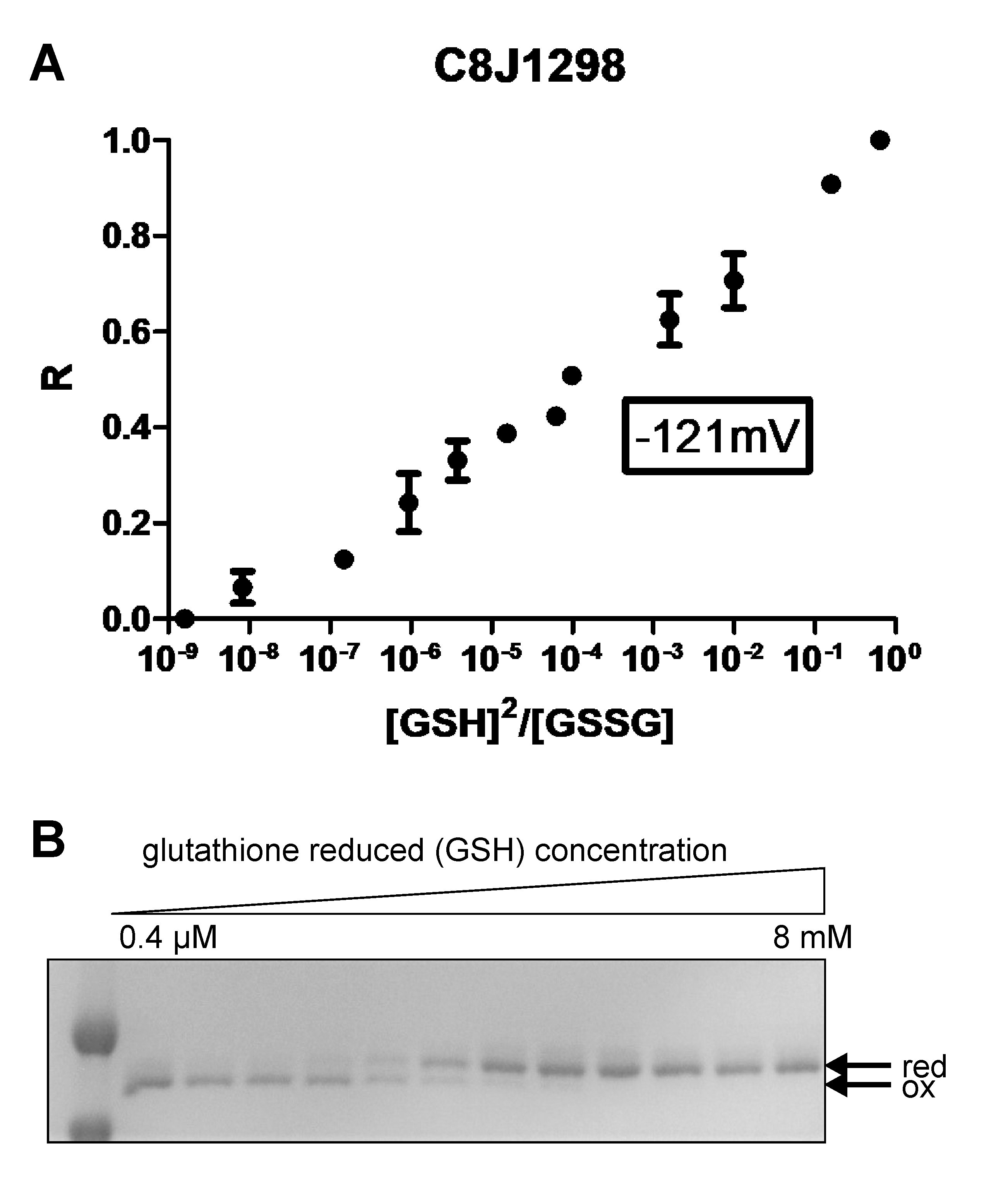

Supplement: S2 Fig — The fraction of reduced (R) C8J_1298 was determined in various glutathione (GSH)/glutathione disulfide (GSSG) ratios using AMS reagent. Fractions (band intensity) of reduced C8J_1298 were determined using Image-Lab (BIO-RAD) after resolving on 14% SDS-PAGE. The standard redox potential was calculated from the Nernst equation using the glutathione standard potential. (A) The bars represent the average of three independent experiments, with two technical repetitions (n = 3). (B) The result of one representative experiment. (TIF) [file pone.0230366.s004.tif]

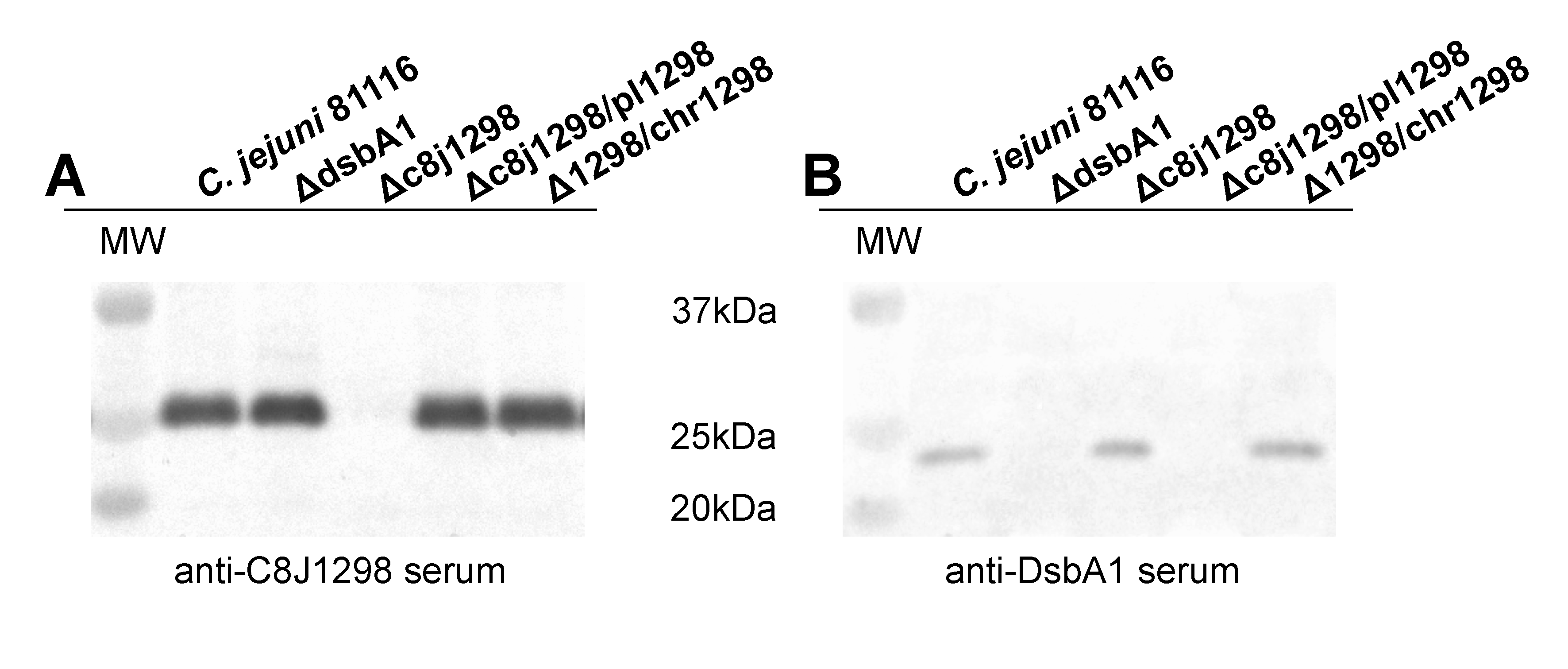

Supplement: S3 Fig — Western blot analysis confirming presence of absence of (A) C8J_1298 and (B) CjDsbA1 in various C. jejuni 81116 strains. Complementation of the Δc8j_1298 mutation restores the presence of C8J_1298. Plasmid complementation (Δc8j_1298/pl1298) of the Δc8j_1298 mutation abolishes production of CjDsbA1. C. jejuni 81116 strains proteins (the whole cell lysate) were separated by 12% SDS-PAGE and electrotransferred onto a nitrocellulose membrane. Specific rabbit sera with antibodies against (A) C8J_1298 or (B) CjDsbA1 were used to verify the absence or presence C8J_1298/CjDsbA1 in C. jejuni 81116 cells. (TIF) [file pone.0230366.s005.tif]

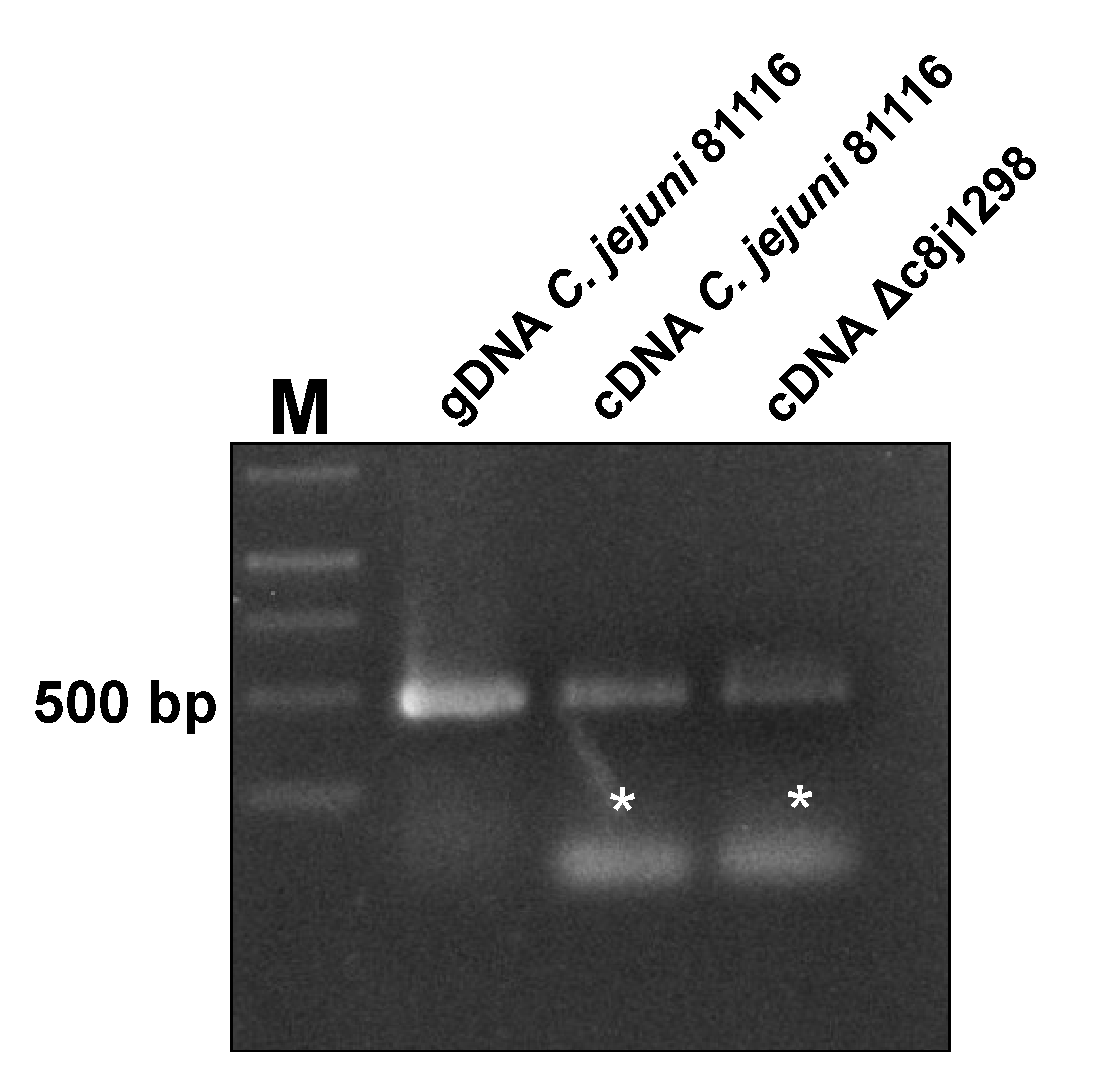

Supplement: S4 Fig — To verify if c8j_1299 transcription is affected by mutation of c8j_1298, total RNA of C. jejuni strains was isolated, contaminating DNA was removed and cDNA was obtained by reverse transcription. C. jejuni 81116 and Δc8j_1298 cDNA was amplified in a standard PCR reaction with primer pair c8j1299-RT–c8j1299-RT2. As a control, C. jejuni 81116 genomic DNA was used. c8j_1299 transcript was present in wild type as well as in Δc8j_1298 mutant cells. (*) unspecific band. (TIF) [file pone.0230366.s006.tif]

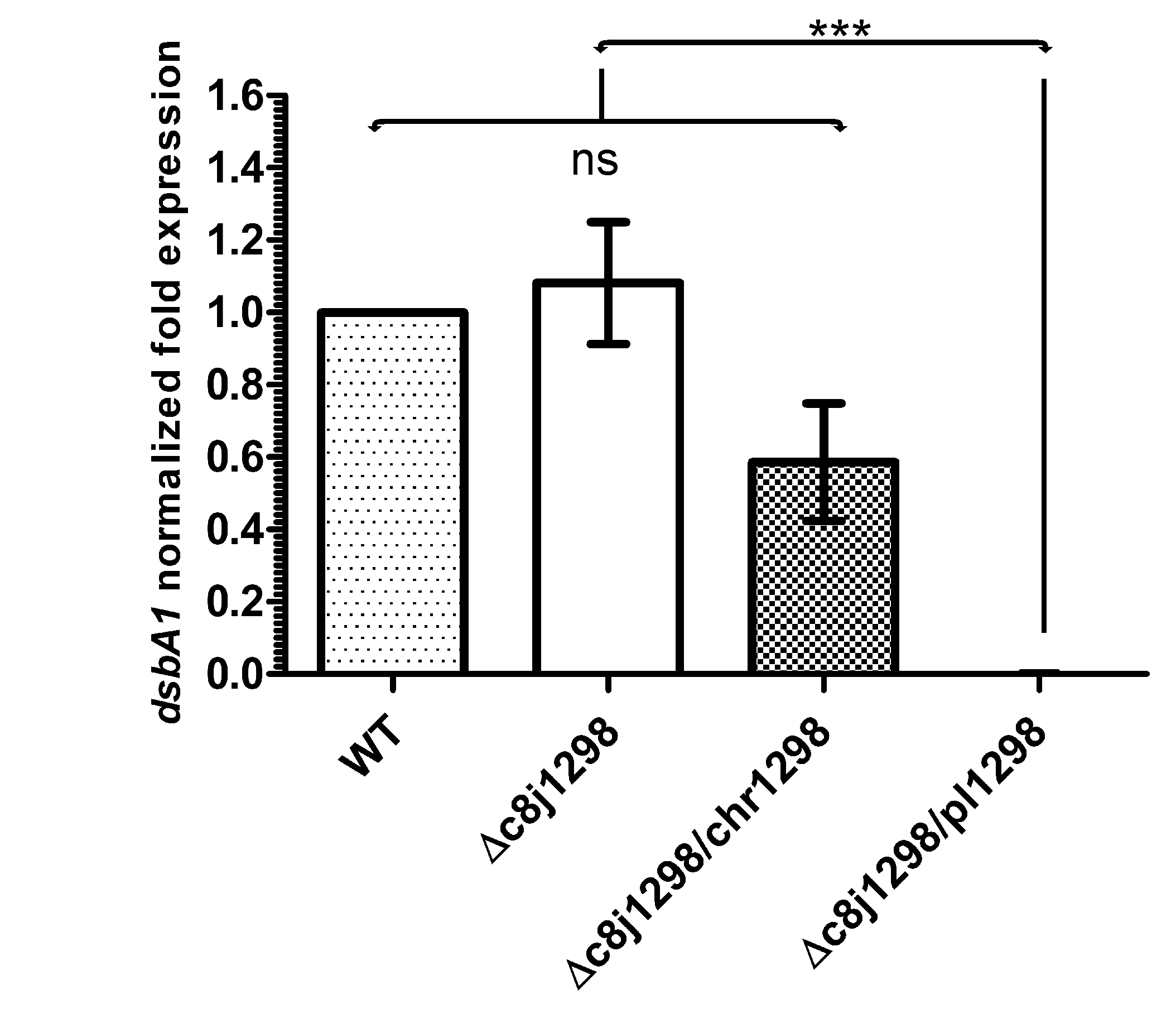

Supplement: S5 Fig — mRNA was isolated from C. jejuni 81116 strains. Results from dsbA1 amplification were normalized using gyrA expression and presented as fold decrease dsbA1. Data presented is the average of three independent experiments, with two technical repetitions (n = 3). p>0,001 ***; p>0,005 **; ns—not significant. (TIF) [file pone.0230366.s007.tif]

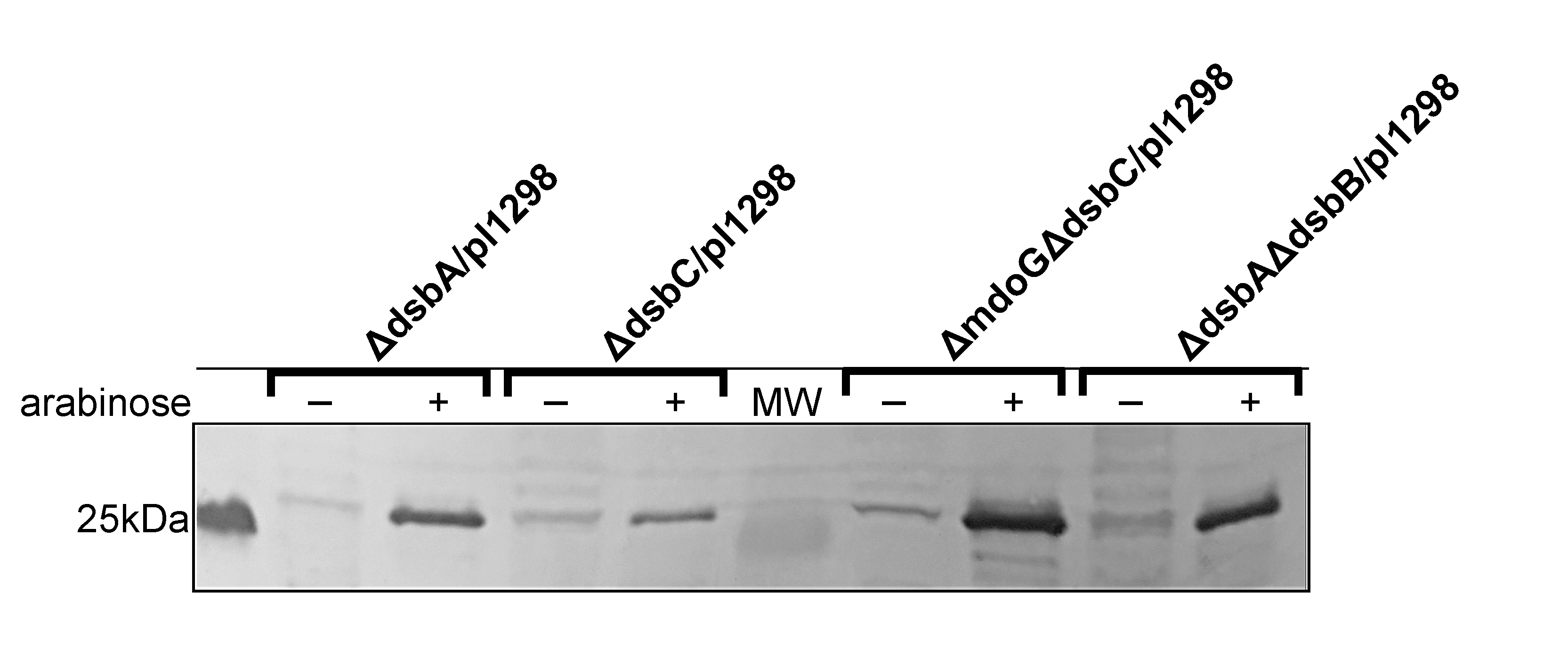

Supplement: S6 Fig — E. coli strains harboring recombinant plasmids were cultured with (+) or without (-) arabinose induction. The c8j_1298 gene was cloned into pMPM-A6 under control of arabinose induced promoter. Proteins (whole cell lysates) from analyzed strains were separated by 12% SDS-PAGE, electrotransferred onto a nitrocellulose membrane and developed with rabbit anti-C8J_1298 serum. (TIF) [file pone.0230366.s008.tif]
