# Supplementary material for: C8J_1298, a bifunctional thiol oxidoreductase of Campylobacter jejuni, affects Dsb (disulfide bond) network functioning
Source: PLoS One. 2020 Mar 23;15(3):e0230366. doi: 10.1371/journal.pone.0230366 (PMC7089426; doi:10.1371/journal.pone.0230366)
Supplement: S1 Text — (DOCX) [file pone.0230366.s012.docx]

**S1 Text. Verification of the CjDsbA1 (AG1 and KBO1) mutant phenotype .**

In this work we wanted to test the interplay between two *C. jejuni* thiol oxidoredoredutases, CjDsbA1 and C8J_1298. As the *c8j_1298* mutated strain (AB1; see main text) and previously generated *dsbA1* deficient non motile strain (AG1) [1] were kanamycin resistant, we generated a new *dsbA1* mutated strain (KBO1) using a chloramphenicol resistance cassette. We transformed a *wt C. jejuni* motile strain with the recombinant plasmid pUWM1458 and checked resulting transformants (forty) in terms of presence the CjDsbA1, CjDsbA2 and C8J_1298 by Western blot analysis. Six of them produced DsbA2 and C8J_1298 but not DsbA1. Additionally all were motile (in contrast to previously described AG1 strain) and produced AstA (arylosulfotransferase), substrate of CjDsbA2. To determine the molecular basis of this inconsistency, a genetic chromosomal complementation approach was used. Recombinant plasmid pUWM1526 was introduced into *C. jejuni dsbA1* Km^r^ (AG1), which resulted in insertion of the wild type *dsbA1* copy into the *c8j_0049* pseudogene. The correctness of this genetic manipulation was confirmed by PCR reaction and by Western blot analysis with rabbit polyclonal anti-DsbA1 antibodies. We found that this genetic manipulation did not restore the strain motility, which strongly indicated that a second-site background mutation was responsible for loss of motility. Also, a double mutated Δ*dsbA1*Δ*dsbA2* strain (KBO2) generated by introducing a *dsbA1* mutation (plasmid pUWM1306) into a strain lacking DsbA2 was motile, and the complementation of the *dsbA2* mutation using pUWM1516 did not result in the loss of motility. These combined data allowed us to conclude that the lack of the DsbA1 thiol oxidoreductase was not responsible for the observed phenotype (loss of motility) and was a consequence of background mutation/s. Thus, the newly constructed Δ*dsbA1* Cm^r^ motile mutant (KBO1) was used in further experiments. Similar observations concerning a high frequency of spontaneous mutation in genes involved in flagella biogenesis has been described by others [2, 3]. It was documented that various types of mutations (SNPs – single nucleotide polymorphism, or INDELs – small insertion and deletions or phase variation changes due to replication error) associated with cell motility and shape occur under standard laboratory conditions and can be introduced at any stage of genetic manipulation. High frequency of this variation is associated with the lack of a mismatch repair system [4, 5].

1. Grabowska AD, Wywial E, Dunin-Horkawicz S, Lasica AM, Wosten MM, Nagy-Staron A, et al. Functional and bioinformatics analysis of two *Campylobacter jejuni* homologs of the thiol-disulfide oxidoreductase, DsbA. PLoS One. 2014;9(9):e106247. Epub 2014/09/03. doi: 10.1371/journal.pone.0106247. PMID: 25181355.

2. de Vries SP, Gupta S, Baig A, L'Heureux J, Pont E, Wolanska DP, et al. Motility defects in *Campylobacter jejuni* defined gene deletion mutants caused by second-site mutations. Microbiology. 2015;161(12):2316-27. Epub 2015/09/20. doi: 10.1099/mic.0.000184. PMID: 26385289.

2. Mohawk KL, Poly F, Sahl JW, Rasko DA, Guerry P. High frequency, spontaneous motA mutations in *Campylobacter jejuni* strain 81-176. PLoS One. 2014;9(2):e88043. Epub 2014/02/22. doi: 10.1371/journal.pone.0088043. PMID: 24558375.

3. Esson D, Mather AE, Scanlan E, Gupta S, de Vries SP, Bailey D, et al. Genomic variations leading to alterations in cell morphology of *Campylobacter* spp. Scientific reports. 2016;6:38303. Epub 2016/12/03. doi: 10.1038/srep38303. PMID: 27910897.

4. Pascoe B, Williams LK, Calland JK, Meric G, Hitchings MD, Dyer M, et al. Domestication of Campylobacter jejuni NCTC 11168. Microbial genomics. 2019;5(7). Epub 2019/07/17. doi: 10.1099/mgen.0.000279. PMID: 31310201.

**pUWM1458** is a suicide plasmid containing coding sequence of *CjdsbA1* disrupted by chloramphenicol resistance cassette (for mutagenesis)

**pUWM 1526** is a suicide plasmid containing *CjdsbA1* gene cloned with own promoter (for chromosomal complementation)

**pUWM1306** is a suicide recombinant plasmid containing coding sequence of *CjdsbA1* disrupted by kanamycin resistance cassette (for mutagenesis)

**pUWM 1516** is a suicide plasmid containing *CjdsbA2* gene cloned with own promoter (for chromosomal complementation)

**Methods:**

**DNA manipulation**

**Double mutant generation** - To generate the double mutant Δ*CjdsbA1*Δ*dsbA2* (KBO2), pUWM1306 was introduced to the *CjdsbA2* deficient strain (AG2).

**Construction of plasmid for chromosomal *dsbA1/dsbA2* complementation experiments -**To obtain constructs for chromosomal complementation by selected gene introduction into the *C. jejuni* *c8j_0049* pseudogene, both arms of the pseudogene were amplified from the *C. jejuni* 81116 genome using primer pairs (c8j0048Sac – c8j0049Not and c8j0049Xho – c8j0050Kpn; S2 Table) designed to create a gap in the pseudogene nucleotide sequence (933 bp). PCR products (1328 bp and 1412 bp) were directionally cloned into pBluescript KSII. Subsequently, chloramphenicol and spectinomycin resistance cassettes (*cat* gene excised from pRY109, spectomycin resistance gene from pNBSpec) were inserted between the pseudogene arms, in an orientation opposite to the inactivated genes. In the space between the arms and the cassette, a few restriction enzyme sites were left to allow cloning genes of interest under their own promoter sequences. Correct construction of novel chromosome complementation plasmids pBC49 and pBS49 was confirmed by sequencing. To confirm correctness of constructed plasmids, control electroporation to *C. jejuni* 81116 was performed. Introduction of the appropriate DNA fragment by double crossing-over into the sequence of pseudogene *c8j_0049* was verified by PCR analysis and sequencing. Next, the nucleotide coding sequence of *CjdsbA1* or *CjdsbA2* with its own promoter was amplified from the *C. jejuni* 81116 genome using primer pairs; C8J0813_Spe – C8J0813_Xho; and C8J0811_Spe – C8J0811_Xho (S2 Table) and cloned into pJet 1.2/blunt vector to form pUWM1506 and pUWM1505, respectively. Using XbaI and NotI restriction enzymes, fragments coding *CjdsbA1or CjdsbA2* with their own promoter sequences were cloned into pBS49, respectively, in the identical transcriptional orientation as the cassettes. Correct construction of plasmids pUWM1526 and pUWM1516 was confirmed by sequencing. Subsequently, the plasmids were introduced by electroporation into appropriate *C. jejuni* 81116 strains. Double cross-over in the sequence of pseudogene *c8j_0049* was verified by PCR analysis and sequencing. Resulting strains were used for the complementation assays.

**Assesment of AstA activity**

Qualitative assays for AstA (arylsulfatase) activity were carried out as described previously [[39](#_ENREF_39), [90](#_ENREF_90)]. Briefly, *C. jejuni* was grown for 16 hours on MH plates supplemented with XS (5-bromo-4-chloro-3-indolylsulfate, 100 mg/ml), a substrate for arylsulfatase, and bacterial colonies were observed for blue color acquisition, which indicates AstA activity. The assays were performed in triplicate (n=3).
