# Supplementary material for: C8J_1298, a bifunctional thiol oxidoreductase of Campylobacter jejuni, affects Dsb (disulfide bond) network functioning
Source: PLoS One. 2020 Mar 23;15(3):e0230366. doi: 10.1371/journal.pone.0230366 (PMC7089426; doi:10.1371/journal.pone.0230366)
Supplement: S1 Raw images — (PDF) [file pone.0230366.s013.pdf]

Fig 2D.

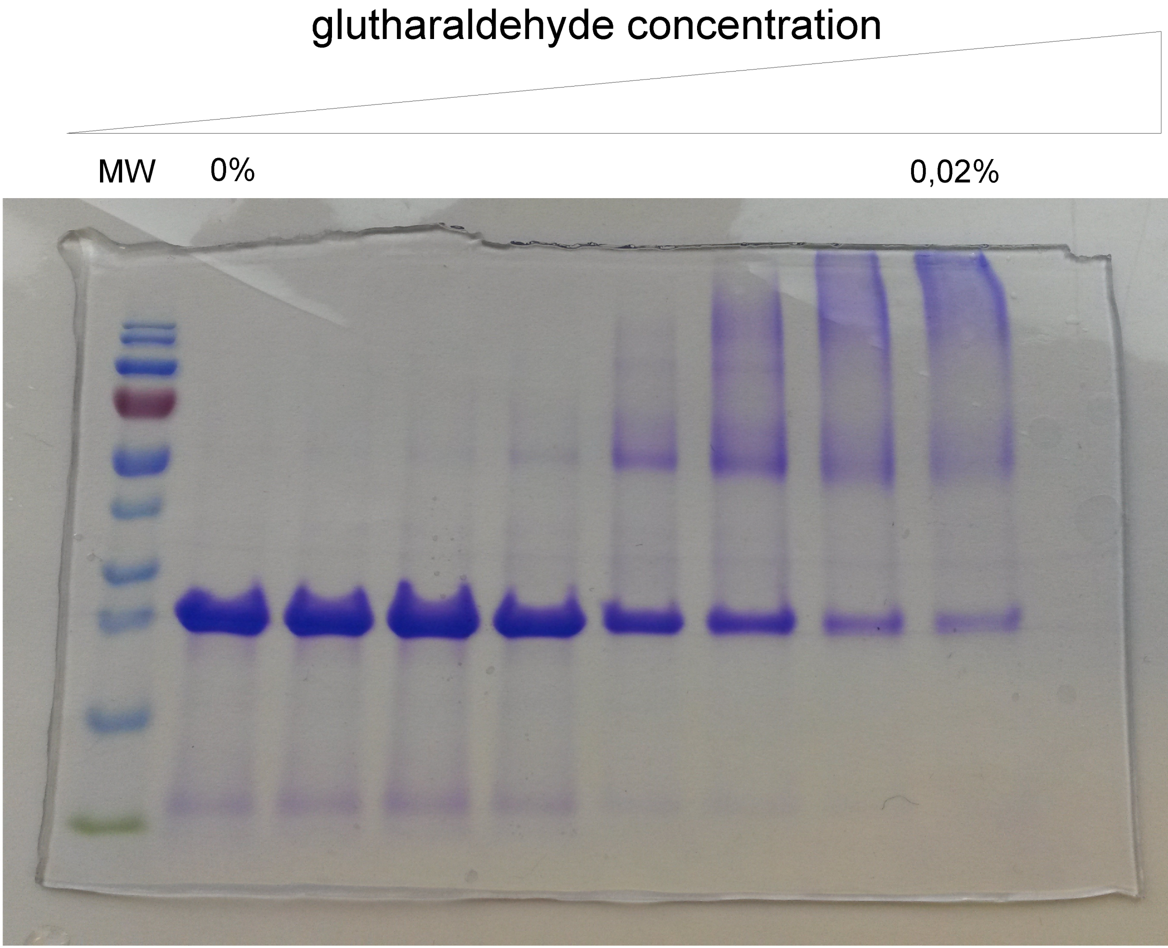

captured using mobile phone

Fig 3A

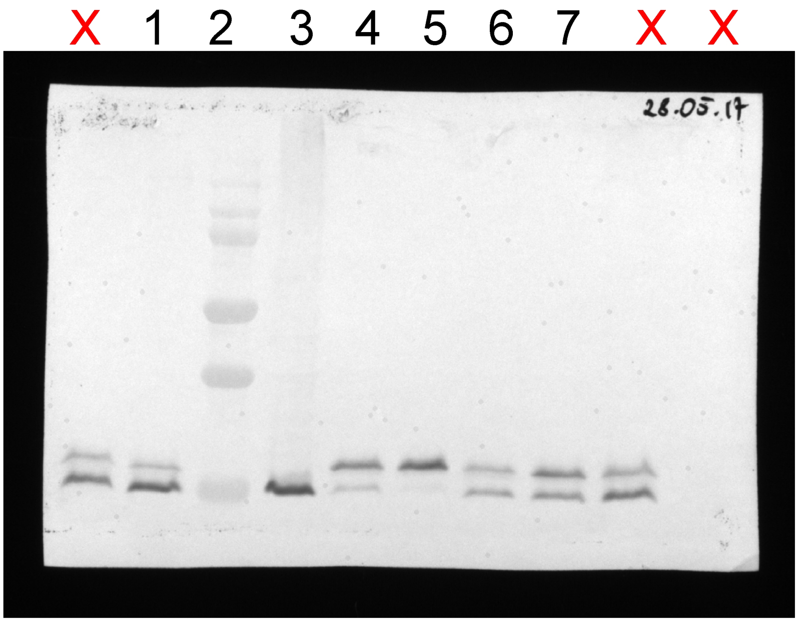

1 - Δ*dsbD* + AMS
2 - MW
3 - WT
4 - WT + AMS
5 - WT +DTT + AMS
6 - Δ*dsbI* + AMS
7 - Δ*dsbB* + AMS

Fig 3B

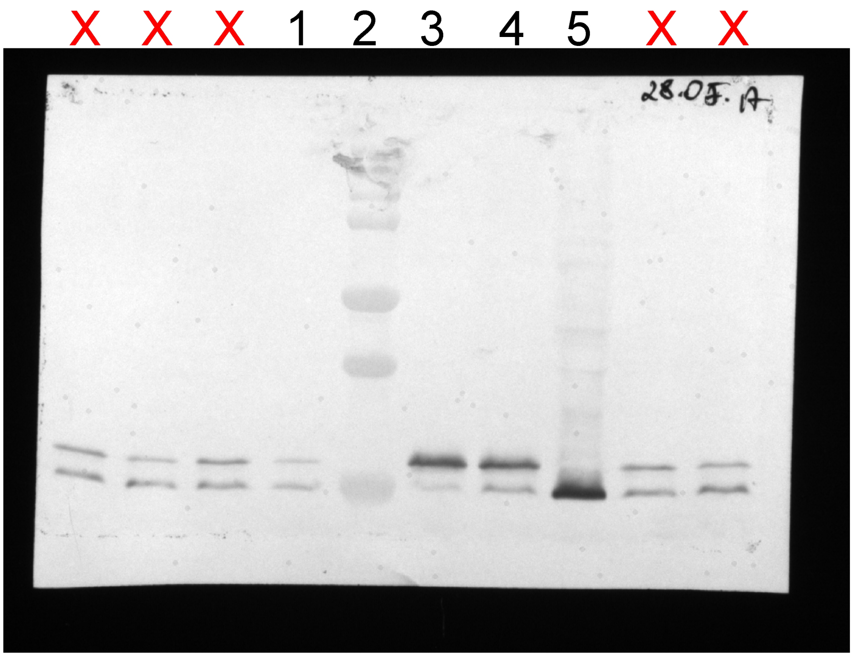

1 - Δ*dsbA1* + AMS
2 - MW
3 - WT+DTT + AMS
4 - WT + AMS
5 - WT

captured using Bio-Rad Gel Doc XR+ system

Fig 4A

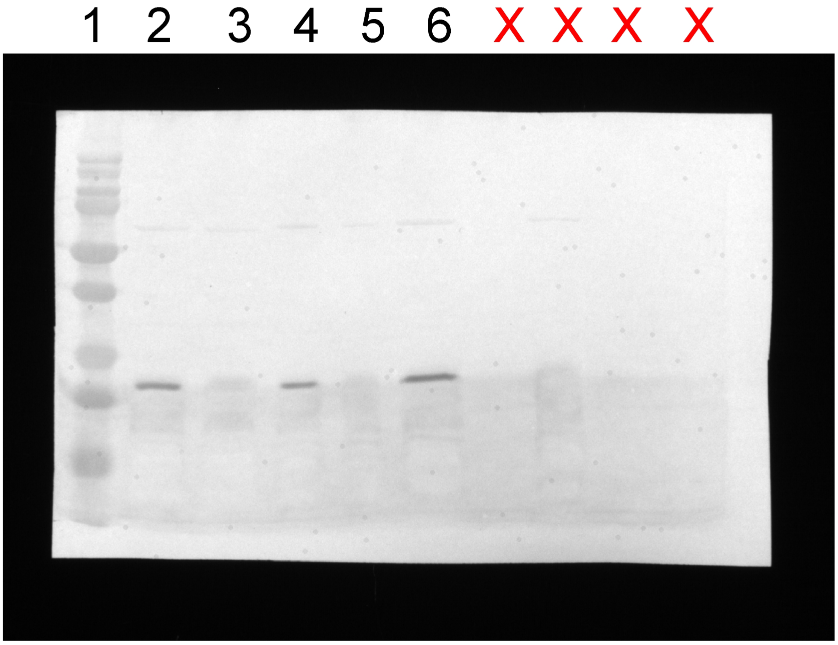

1 - Molecular weight marker
2 - *C. jejuni* 81116 WT
3 - Δ*dsbA1*
4 - Δ*c8j_1298*
5 - Δ*dsbA1*Δ*c8j_1298*
6 - Δ*dsbA1*Δ*c8j_1298*/chrA1

Fig 4B

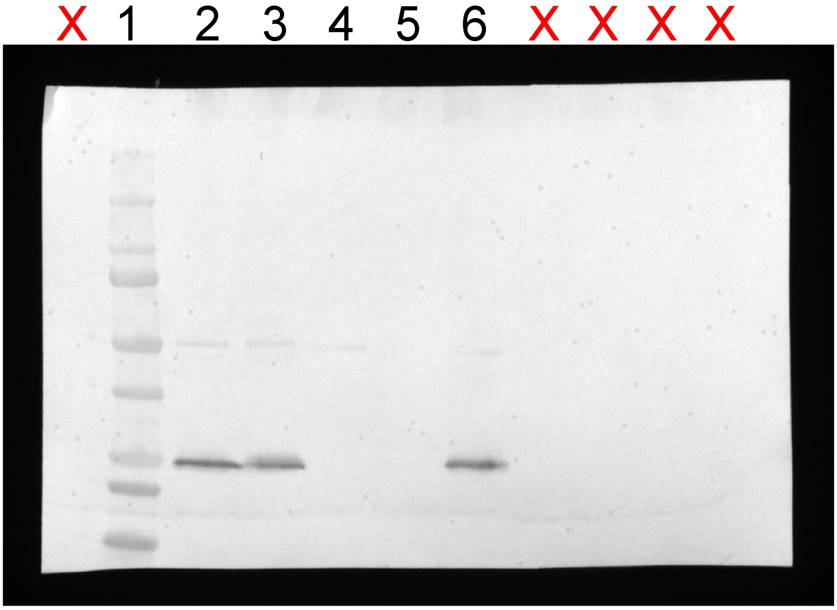

1 - Molecular weight marker
2 - *C. jejuni* 81116 WT
3 - Δ*dsbA1*
4 - Δ*c8j_1298*
5 - Δ*dsbA1*Δ*c8j_1298*
6 - Δ*dsbA1*Δ*c8j_1298*/chr1298

captured using Bio-Rad Gel Doc XR+ system

Fig 7A

1 2 3 4 5 6 7 8 9

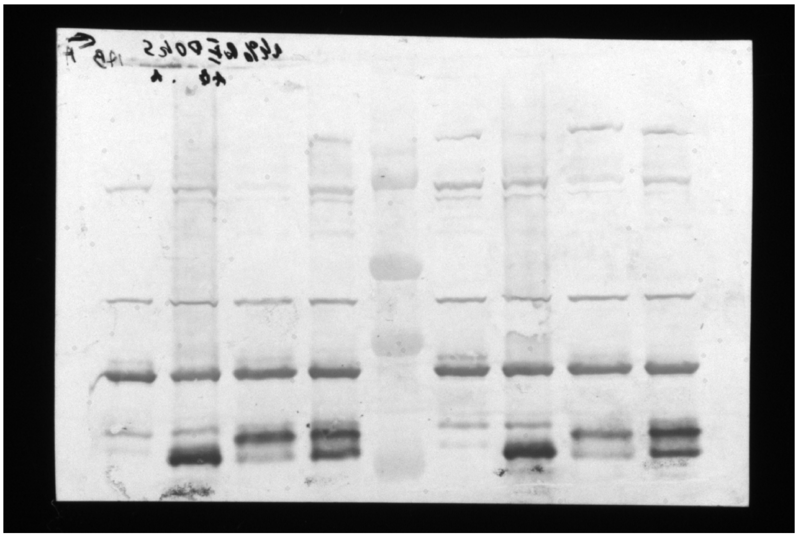

1 - Δ*dsbA*
2 - Δ*dsbA*/pl1298
3 - Δ*dsbA*/pl1298 +DTT + AMS
4 - Δ*dsbA*/pl1298 + AMS
5 - Molecular weight marker
6 - Δ*dsbA*Δ*dsbB*
7 - Δ*dsbA*Δ*dsbB/*pl1298
8 - Δ*dsbA*Δ*dsbB/*pl1298 +DTT + AMS
9 - Δ*dsbA*Δ*dsbB/*pl1298 + AMS

Fig 7B

X 1 2 3 4 5 X X X

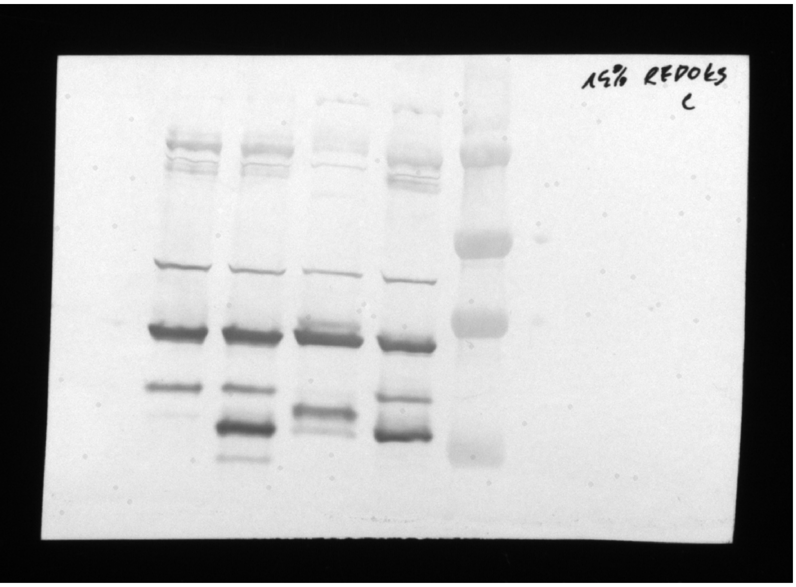

1 - Δ*dsbC*
2 - Δ*dsbC*/pl1298
3 - Δ*dsbC*/pl1298 +DTT + AMS
4 - Δ*dsbC*/pl1298 + AMS
5 - Molecular weight marker

captured using Bio-Rad Gel Doc XR+ system

S2 Fig

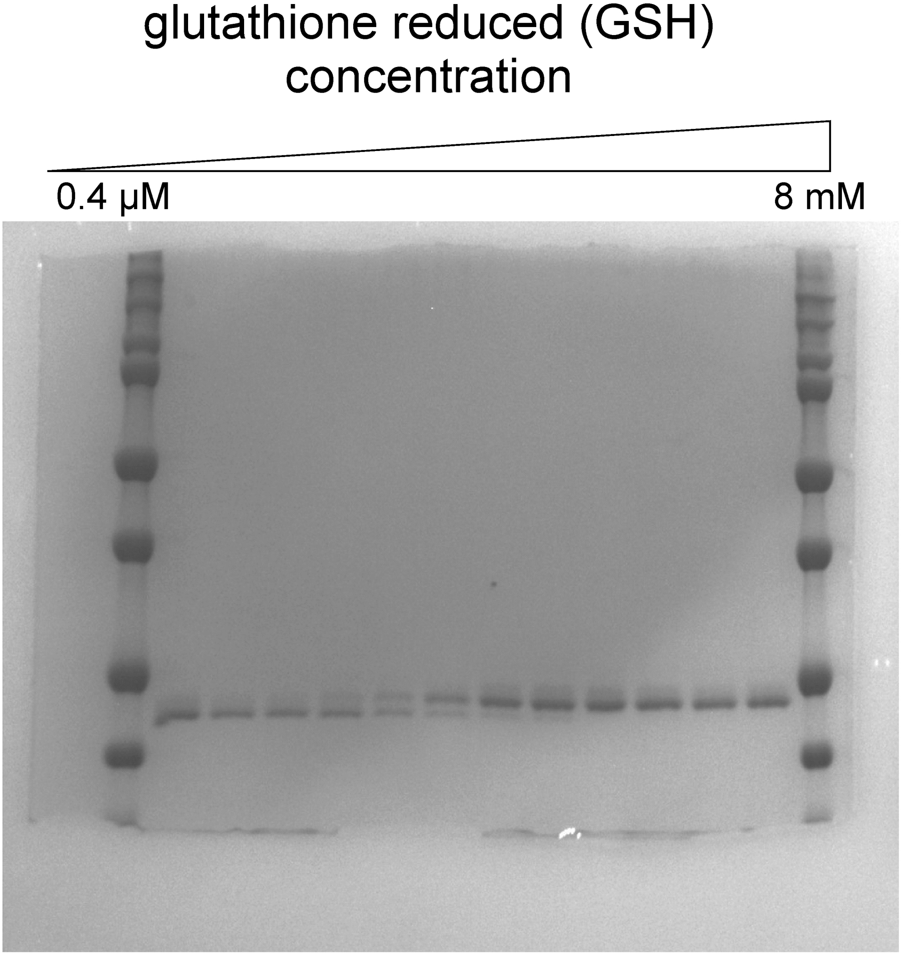

captured using Bio-Rad Gel Doc XR+ system

S3A Fig

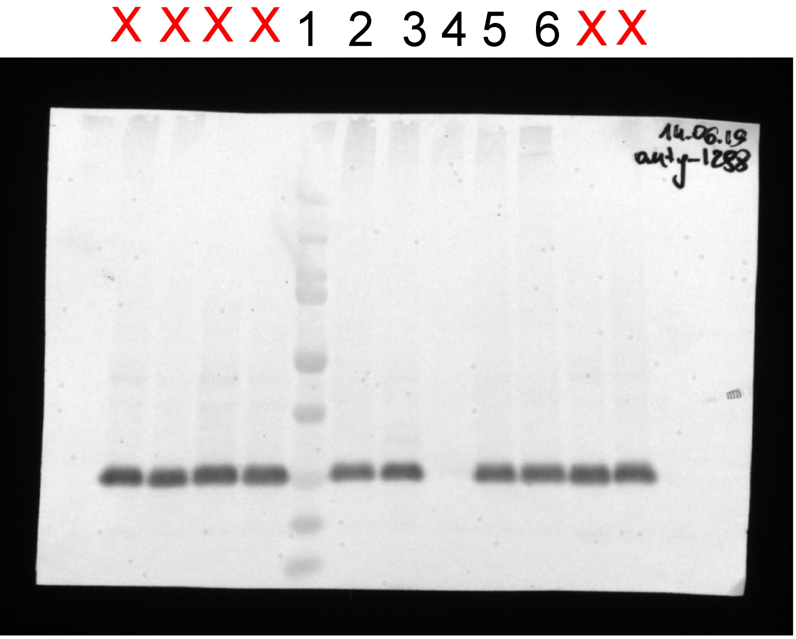

1 - Molecular weight marker
2 - *C. jejuni* 81116 WT
3 - Δ*dsbA1*
4 - Δ*c8j_1298*
5 - Δ*c8j_1298*/pl1298
6 - Δ*c8j_1298*/chr1298

S3B Fig

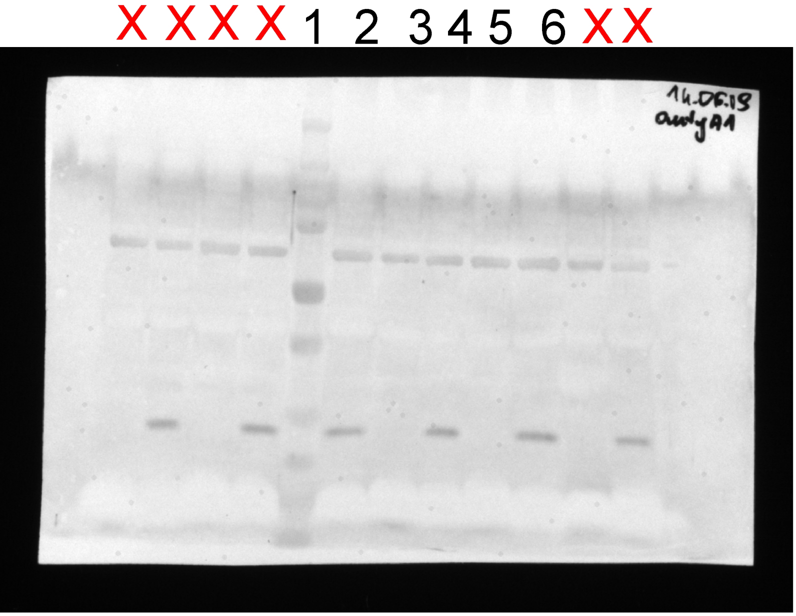

1 - Molecular weight marker
2 - *C. jejuni* 81116 WT
3 - Δ*dsbA1*
4 - Δ*c8j_1298*
5 - Δ*c8j_1298*/pl1298
6 - Δ*c8j_1298*/chr1298

captured using Bio-Rad Gel Doc XR+ system

S4 Fig

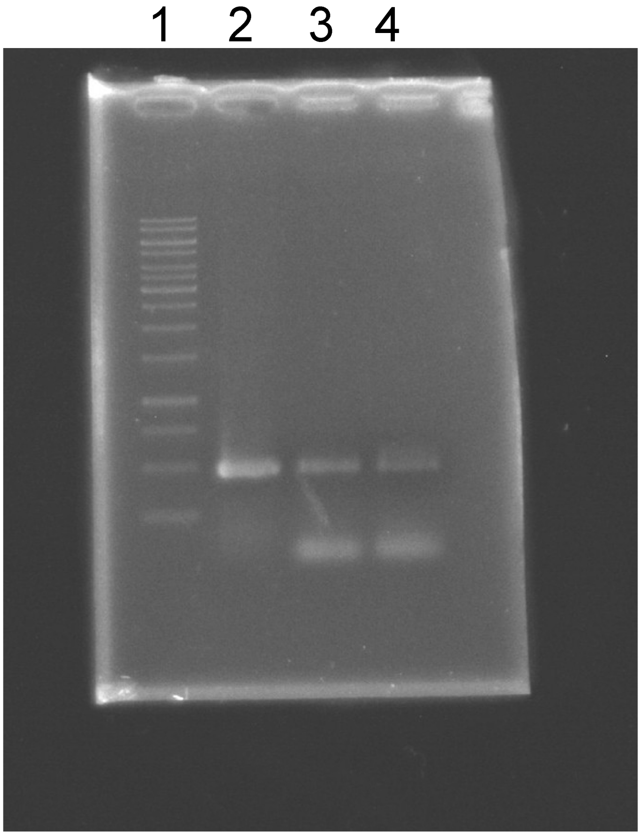

1 - Molecular weight marker
2 - gDNA *C. jejuni* 81116 WT
3 - cDNA *C. jejuni* 81116 WT
4 - cDNA Δ*c8j_1298*

captured using Bio-Rad Gel Doc XR+ system

S6 Fig

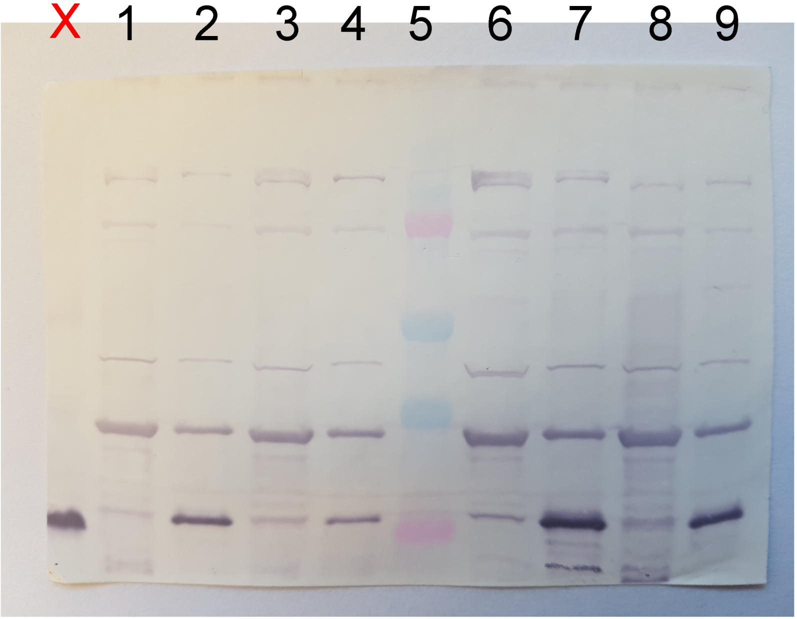

1 - Δ*dsbA*/pl1298 - ara
2 - Δ*dsbA*/pl1298 + ara
3 - Δ*dsbC*/pl1298 - ara
4 - Δ*dsbC*/pl1298 + ara
5 - Molecular weight marker
6 - Δ*mdo*GΔ*dsbC*/pl1298 - ara
7 - Δ*mdo*GΔ*dsbC*/pl1298 + ara
8 - Δ*dsbA*Δ*dsbB*/pl1298 - ara
9 - Δ*dsbA*Δ*dsbB*/pl1298 + ara

captured using mobile phone
